# The German emotion beliefs questionnaire: factorial validity, measurement invariance, and descriptive reference values in a population-representative sample

**DOI:** 10.1186/s40359-026-05837-z

**Published:** 2026-10-23

**Authors:** Jule Romea Lammers, Elmar Brähler, Ansgar Koechel, Rupert Conrad, Markus Ramm

**Affiliations:** 1 Department of Psychosomatic Medicine and Psychotherapy, University Hospital Münster, Albert-Schweitzer- Campus 1, 48149 Münster, Germany; 2 Department of Psychosomatic Medicine and Psychotherapy, University Medical Center of the Johannes Gutenberg-University Mainz, Mainz, Germany; 3 Clinic and Polyclinic for Psychosomatic Medicine and Psychotherapy, Rostock University Medical Center, Rostock, Germany

**Keywords:** Emotion beliefs, Emotion regulation, General population, Psychopathology

## Abstract

**Background:**

Beliefs about emotions are considered a central mechanism of emotion regulation and mental health [1]. The 16-item Emotion Beliefs Questionnaire (EBQ; [2]) assesses beliefs about the usefulness and controllability of positive and negative emotions. While the original study supported a three-factor model, subsequent population-based studies favored the theoretically proposed four-factor structure. Although two German-language adaptations exist, neither has been validated in a population-representative sample, and descriptive reference values, evidence for measurement invariance across key sociodemographic groups, and systematic investigations of sociodemographic correlates of emotion beliefs are still lacking.

**Methods:**

The German version of the EBQ [3] was administered to a representative sample of the German general population (*N* = 2368; mean age = 50.3; SD = 17.13; range: 18–93; 44.7% men, 55.2% women). Confirmatory factor analyses (MLR) were conducted to compare competing three- and four-factor models. Measurement invariance across gender, age, and educational level was examined and descriptive reference values were calculated.

**Results:**

Although the four-factor model fit better than the three-factor solution on all indices examined, the two controllability factors were very highly correlated (*r* = .90) and showed limited discriminant validity, indicating a largely valence-independent controllability dimension. Bifactor analyses supported interpretation of the total score (ω_H = 0.792, ECV = 0.603), and the EBQ further demonstrated good psychometric properties. Measurement invariance was supported up to the strict level across age and gender, and approximately at the scalar level across education. Educational attainment showed the most consistent associations with emotion beliefs across the total score and all subscales. Descriptive percentile reference values are provided by age group and gender.

**Conclusions:**

The German EBQ demonstrated satisfactory psychometric properties in a representative adult sample. Population-based descriptive reference values may facilitate research comparisons and preliminary clinical contextualization of individual scores. The findings support a largely valence-independent controllability dimension and highlight educational attainment as a consistent sociodemographic correlate of emotion beliefs.

**Supplementary Information:**

The online version contains supplementary material available at https://doi.org/10.1186/s40359-026-05837-z .

## Background

 Emotional experiences constitute a fundamental and pervasive component of human functioning, with evidence indicating that individuals report being in an emotional state up to 90% of the time [4]. Given this prevalence, it is hardly surprising that people develop varied beliefs on what emotions are, what purpose they serve, and to what extent they can be controlled [5, 6]. According to Ford and Gross [1], these beliefs can be conceptualized along two primary dimensions: the perceived *goodness* of emotions (i.e., to what extent emotions are considered valuable or helpful) and their perceived *controllability* (i.e., to what extent emotions can be consciously altered). From a clinical perspective, beliefs about emotions are highly relevant, as they shape both the motivation and implementation of emotion regulation processes [1]. Beliefs about the *goodness* (later operationalized as *usefulness* by [2]) of emotions shape the appraisal of emotional states as desirable or undesirable and thus influence which regulation strategies individuals are motivated to employ, for instance, whether negative emotions are accepted or suppressed. Research shows that perceiving emotions as undesirable promotes avoidance-based strategies [7]. Beliefs about controllability, in turn, determine whether regulation is initiated, how much effort is invested, and whether regulation attempts are sustained or abandoned. Empirically, beliefs regarding the uncontrollability of emotions are associated with less frequent use of adaptive strategies such as cognitive reappraisal and lower self-efficacy in emotion regulation [8, 9]. This influence on emotion regulation represents a central mechanism through which emotional beliefs are linked to mental health. Targeting dysfunctional emotion beliefs represents a promising avenue for clinical interventions, as modifying these beliefs may facilitate the acquisition and effective use of adaptive regulation strategies and, in turn, improve mental health [10, 11].

To provide a psychometric assessment of the belief dimensions proposed by Ford and Gross [1], Becerra et al., [2] developed the Emotion Beliefs Questionnaire (EBQ). Earlier scales, for example the Implicit Theories of Emotions Scale (ITES; [12]), the Beliefs about Emotions Scale (BES; [13]), or the Emotion and Regulation Beliefs Scale (ERBS; [14]) did not simultaneously meet all three key criteria outlined by the authors: (1) the distinction between beliefs about controllability and usefulness, (2) assessment at the superordinate level (i.e., beliefs about emotions in general rather than about one’s own emotion regulation self-efficacy), and (3) differentiation between positive and negative emotions. The EBQ addresses these gaps by assessing beliefs about the controllability and usefulness of positive and negative emotions across four subscales, with higher scores reflecting stronger endorsement of maladaptive beliefs. However, in the original study using an Australian sample (*N* = 161), the four-factor model showed signs of empirical underidentification, as the two controllability subscales were perfectly correlated, rendering a three-factor solution the best-fitting viable model. Accordingly, the results indicated a three-factor structure consisting of General Controllability, Negative Usefulness, and Positive Usefulness, with an overarching general factor [2]. In contrast to these initial findings, international validation studies in American [15], Iranian [16], Italian [17] and Polish [18] samples provide support for the originally proposed four-factor model based on fit indices. The EBQ has been shown to demonstrate acceptable to good internal consistency [2, 15–18], satisfactory test-retest reliability [16] as well as measurement invariance across age, education and gender categories [15–18]. EBQ scores show the expected associations with difficulties in emotion regulation [17], emotional reactivity [18] and lesser use of adaptive emotion regulation strategies [19]. Across studies, higher EBQ scores are consistently associated with elevated symptoms of depression, anxiety, and stress [15, 17, 18, 20]. Mazidi et al., [21] used the EBQ to identify six emotion belief profiles in a clinical sample, with the two most maladaptive profiles exhibiting the greatest psychopathology and emotion regulation difficulties.

Two independent German-language adaptations of the Emotion Beliefs Questionnaire (EBQ) have been developed using different translation approaches. The first adaptation by Biel et al., [3] employed a forward-backward translation procedure conducted by native German speakers. In a sample of psychotherapy trainees (*N* = 104), the authors replicated the original three-factor structure using exploratory factor analysis. Internal consistency was good for the total scale (α = 0.87) and the General Controllability subscale (α = 0.85), and acceptable for Negative Usefulness (α = 0.76) and Positive Usefulness (α = 0.70). Evidence for concurrent validity was provided by associations with emotion acceptance and symptoms of depression, anxiety, and stress. Beyond its initial validation, the Biel et al., version has already demonstrated clinical relevance, supporting its conceptualization, from a psychodynamic perspective, as a measure of affective mentalization: In a prospective panel study, baseline EBQ scores predicted somatic symptom-related distress at follow-up beyond autoregressive effects, with findings replicated in psychosomatic outpatient samples [22]. Further evidence comes from a multi-study investigation linking EBQ-assessed affective mentalization to depression. Higher EBQ scores were associated with greater depression severity in a large psychosomatic sample, and longitudinal analyses in a general population sample indicated that baseline EBQ scores predicted subsequent depressive symptoms. Additionally, an experimental study showed that a targeted intervention improved affective mentalization and reduced induced depressive states [23]. Together, these findings support the clinical sensitivity and predictive validity of the Biel et al., adaptation.

A second German validation by Gutzweiler and Grüning [24], based on an independent translation, examined a larger sample of students and young adults (*N* = 348) using confirmatory factor analysis. Results favored a four-factor model, which outperformed competing models, including the three-factor model. Internal consistency ranged from acceptable to good (α = 0.68-0.79; ω = 0.70-0.80), and construct validity was supported by expected associations with emotion regulation, emotional reactivity, self-efficacy, and psychopathology. The measure demonstrated largely strict measurement invariance across gender as well as across levels of emotional reactivity and self-efficacy [24]. Taken together, the two German adaptations reflect the broader pattern of cross-national discrepancies in EBQ factor structure. While the exploratory three-factor solution reported by Biel et al., [3] aligns with the original Australian findings, the confirmatory four-factor solution identified by Gutzweiler and Grüning [24] is consistent with subsequent validations in diverse cultural contexts.

Several limitations remain with respect to the Biel et al., version, despite its promising clinical applications. The initial validation relied on a small, young, and predominantly female convenience sample, limiting generalizability to the broader German population. Crucially, population-based reference values are currently lacking, which restricts the interpretability of individual scores. Reference values are essential for the meaningful (clinical) application of self-report instruments, as they allow individual scores to be interpreted relative to a defined reference population. While such norms have been established for the English [15] and Polish [18] versions, no population-based reference values are currently available for the German EBQ. In addition, measurement invariance across key sociodemographic variables remains insufficiently examined in German samples as German validation studies have not systematically tested invariance across age or educational levels. This gap is theoretically relevant, as emotion-related beliefs and regulation strategies are known to vary across the lifespan. According to socioemotional selectivity theory, age-associated shifts in motivational priorities influence emotion regulation goals and preferences [25], potentially affecting how EBQ items are interpreted across age groups. Furthermore, the role of sociodemographic factors such as education and socioeconomic status (SES) in shaping emotion beliefs remains largely unexplored. Existing evidence suggests that lower SES is associated with greater anxiety and emotional distress, and that SES moderates the effectiveness of adaptive emotion regulation strategies such as cognitive reappraisal [26]. Given the close link between emotion beliefs, appraisal processes, and perceived regulatory utility [1], it is plausible that SES-related differences in regulatory outcomes are partly reflected in differences in emotion beliefs. However, this question has not yet been systematically addressed within the EBQ framework.

Against this background, the present study aims to further validate the German EBQ [3] in a large, population-representative sample of German-speaking adults. Specifically, the study seeks to (1) examine the factorial validity by comparing three- and four-factor models using confirmatory factor analysis, (2) test measurement invariance across key sociodemographic variables, including gender, age, and educational background, (3) investigate associations between emotion beliefs and sociodemographic characteristics, and (4) provide population-based reference values to support both research and clinical application by enabling the interpretation of individual scores relative to the general population.

## Methods

### Subjects

Data were acquired between March and June 2025 by a survey research institute (USUMA, Berlin, Germany) as part of a broader German household survey. A multistage random sampling procedure was used: proportionally to the distribution of private households, Germany was divided into 258 sampling areas covering all regions. Within these areas, households were selected using a random-route procedure, and target persons within households were randomly selected. In total, *N* = 6663 households were contacted, approaching a representative sample of the German general population. *N* = 2556 subjects aged ≥ 16 years gave written informed consent to participate, resulting in *N* = 2522 analyzable interviews (effective response rate: 37.9%). The survey consisted of an interviewer-administered section designed to collect sociodemographic data and a self-administered questionnaire. In our analysis, participants < 18 years (*N* = 37) were excluded. Furthermore, subjects with missing items in the EBQ (*N* = 117) were also excluded, resulting in a final sample of *N* = 2368.

### Measure

The Emotion Beliefs Questionnaire [2] is a 16-item self-report questionnaire assessing beliefs about the controllability (e.g., whether emotions can be regulated) and usefulness (e.g., whether emotions are helpful or harmful) of positive and negative emotions across four subscales. Items are rated on a seven-point Likert scale (1 = strongly disagree to 7 = strongly agree). Higher scores indicate stronger endorsement of maladaptive beliefs regarding the uncontrollability and lack of usefulness of emotions. Subscale scores and a total score are calculated by summing the corresponding items, resulting in a total EBQ score ranging from 16 to 112. The items of the EBQ and their corresponding subscales are presented in Table 2. For the present study, the German version of the EBQ validated by Biel et al., [3] was used. The translation followed the European Social Survey’s Translation Guidelines, involving multiple independent translations. The German EBQ items are provided in Supplement 1.

### Statistical analysis

Statistical analysis was carried out with R version 4.5.1. (R Core Team [27]), primarily using lavaan, semTools, psych, car, lmtest, and sandwich. To examine the factor structure of the Emotion Beliefs Questionnaire, confirmatory factor analyses (CFA) using a robust maximum likelihood estimator (MLR) were conducted, in which a one-factor, a three-factor, and a four-factor model were tested. The four-factor model comprised the dimensions Negative Controllability, Positive Controllability, Negative Usefulness, and Positive Usefulness, each with four items per factor. In the three-factor model, the two controllability dimensions were combined into a single factor, while the one-factor model assumed a general factor across all items. Model fit was assessed using scaled Chi-square (χ²), robust Root Mean Square Error of Approximation (RMSEA), robust Comparative Fit Index (CFI), robust Tucker-Lewis Index (TLI), Standardized Root Mean Square Residual (SRMR) and Bayesian information criterion (BIC). Model fit was evaluated according to established cut-off criteria [28]. Nested models were compared using the Satorra-Bentler scaled difference test [29]. Internal consistency was assessed using Cronbach’s alpha (α) and McDonald’s omega (ω_total). To evaluate the interpretability of the EBQ total score despite the multidimensional factor structure, we additionally fitted a bifactor model comprising one general factor loading on all 16 items and three orthogonal group factors (Controllability, Negative Usefulness, Positive Usefulness) using MLR. Omega hierarchical (ωH), explained common variance (ECV), percentage of uncontaminated correlations (PUC), and factor-score determinacy (FD) were computed from the standardized solution to assess the strength and interpretability of the general factor. Omega hierarchical subscale (ωHS) was calculated to quantify reliable subscale-specific variance beyond the general factor [30]. The corresponding ωH and ωHS coefficients were additionally calculated from the unstandardized bifactor solution for the unweighted raw-item sum scores used in the descriptive reference values and regression analyses. As a robustness check, we additionally fitted a bifactor model with four orthogonal group factors, separating Negative and Positive Controllability. Following established recommendations, ωH values of 0.70-0.80 or higher [31] and FD ≥ 0.90 were considered indicative of a well-defined general factor. ECV and PUC were interpreted jointly rather than against fixed cut-offs: Reise et al., [32] proposed that when PUC falls below 0.80, ECV > 0.60 in combination with ωH > 0.70 indicates that the degree of multidimensionality present does not preclude interpreting the instrument as primarily reflecting a single common dimension.

Discriminant validity of the EBQ dimensions was assessed using latent factor intercorrelations, the Fornell-Larcker criterion, and the heterotrait-monotrait ratio (HTMT; [33]). The Fornell-Larcker criterion compares the average variance extracted (AVE, the mean proportion of item variance accounted for by its factor) with the correlations between factors. Discriminant validity is supported when the square root of the AVE for each factor exceeds its correlations with all other factors, indicating that a factor shares more variance with its own indicators than with any other construct. The HTMT estimates the correlation two constructs would show if both were measured without error, by relating the average correlation of items across constructs to the average correlation of items within constructs. Lower values indicate greater discriminant validity, values below 0.85 were considered indicative of adequate discriminant validity [33].

Descriptive reference values for the EBQ total score were derived using the raw sum scores. Percentiles were calculated for the total sample and stratified by gender and age group. Measurement invariance across gender, age, and education was examined sequentially by fitting configural, metric, scalar, and strict models using MLR. Robust CFI and RMSEA and the SRMR were used to evaluate model fit. Measurement invariance was primarily evaluated using changes in CFI and RMSEA, with decreases in CFI of no more than 0.010 and increases in RMSEA of no more than 0.015 considered indicative of invariance [34, 35]. Because education-group sizes were highly unequal and two groups were small, Chen’s [34] more stringent criteria (ΔCFI ≤ 0.005 in combination with ΔRMSEA ≤ 0.010 or ΔSRMR ≤ 0.010) were additionally considered as a conservative sensitivity benchmark. For education, we further examined model fit separately within each group and conducted item-level score-test diagnostics of the equality constraints.

Because several Positive Usefulness items were strongly skewed and showed pronounced floor effects, sensitivity analyses were conducted. The CFA models were re-estimated treating the seven-point EBQ items as ordered categorical indicators using WLSMV. Measurement invariance analyses were likewise repeated under the ordinal specification through the scalar level, testing equality of thresholds, factor loadings, and intercepts across groups following Wu and Estabrook [36]. Where necessary, adjacent upper response categories were collapsed consistently across groups to address unobserved or sparsely populated categories.

Multiple linear regression analyses examined associations of age, gender, educational attainment, household income, and marital status with the EBQ total score and the three subscale scores (Controllability, Negative Usefulness, and Positive Usefulness). All four models were estimated in the same complete-case sample (*N* = 2335). Age was mean-centered at 50.42 years. Men, basic school graduation, household income below €1250, and married and living with partner served as reference categories. For each outcome, we report unstandardized coefficients (B) with 95% confidence intervals and standardized coefficients. For age, coefficients were fully standardized as B × SDx/SDy. For categorical predictors, coefficients were standardized with respect to the outcome only (B/SDy) and therefore represent outcome-standardized dummy-coded contrasts. The dummy-coded predictors themselves remained unstandardized. Model fit was summarized using R² and adjusted R². Omnibus tests were conducted for each complete predictor block, with partial R² and ΔR² quantifying its unique contribution after adjustment for the remaining predictors. Benjamini-Hochberg false discovery rate correction was applied separately within each EBQ outcome, with the 14 individual non-intercept coefficients forming one correction family and the five omnibus block tests a second. Outcome-specific correction was used because the EBQ total score is composed of the three subscale scores and the corresponding analyses therefore represent closely related, structurally overlapping sets of tests. As a more conservative sensitivity analysis, the Benjamini-Hochberg correction was additionally applied across all four outcomes jointly (56 coefficient tests and 20 omnibus tests). Bivariate associations were examined to distinguish unadjusted from mutually adjusted associations: Pearson correlations were calculated for age, and separate unadjusted linear models with omnibus F tests were fitted for the categorical predictors. Regression assumptions were evaluated using residual diagnostic plots, generalized variance inflation factors, the Breusch-Pagan test, models including a quadratic age term, and influence diagnostics based on standardized residuals, leverage, and Cook’s distance. As a further sensitivity analysis, HC3 heteroskedasticity-consistent inference was used for coefficient and omnibus tests.

## Results

### Sample Characteristics

Table 1 shows the sociodemographic characteristics of the analyzed sample. The sample consisted of 44.7% men, 55.2% women, and 0.1% diverse gendered individuals. The mean age was 50.32 years (SD = 17.13; range: 18–93 years).

**Table 1:** Sample characteristics

	Total (*N* = 2368)	Men (*N* = 1059)	Women (*N* = 1306)
**Age,** M (SD)	50.32 (17.13)	50.44 (17.09)	50.26 (17.17)
**Age groups**	N (%)	N (%)	N (%)
< 30 years	356 (15.0)	163 (15.4)	193 (14.8)
30–39 years	367 (15.5)	158 (14.9)	207 (15.8)
40–49 years	399 (16.8)	163 (15.4)	235 (18.0)
50–59 years	454 (19.2)	211 (19.9)	243 (18.6)
60–69 years	452 (19.1)	216 (20.4)	236 (18.1)
≥ 70 years	340 (14.4)	148 (14.0)	192 (14.7)
**Marital status**			
Married (living with partner)	1086 (46.1)	498 (47.2)	588 (45.2)
Married (living without partner)	51 (2.2)	18 (1.7)	33 (2.5)
Unmarried (in a relationship)	268 (11.4)	117 (11.1)	150 (11.5)
Unmarried (not in a relationship)	439 (18.6)	235 (22.3)	203 (15.6)
Divorced	316 (13.4)	136 (12.9)	179 (13.8)
Widowed	197 (8.4)	50 (4.7)	147 (11.3)
**Education**			
Without graduation	60 (2.5)	30 (2.8)	30 (2.3)
Basic school graduation	549 (23.2)	267 (25.3)	281 (21.5)
Middle maturity	989 (41.8)	378 (35.8)	610 (46.7)
Technical school	99 (4.2)	46 (4.4)	53 (4.1)
A-level	357 (15.1)	186 (17.6)	170 (13.0)
University graduation	307 (13.0)	150 (14.2)	157 (12.0)
Currently in school	5 (0.2)	0 (0.0)	5 (0.4)
**Occupational status**			
Regularly employed	1431 (60.5)	668 (63.2)	760 (58.3)
Currently unemployed/short-time work/ marginally employed	144 (6.1)	55 (5.2)	89 (6.8)
Schooling/professional training	103 (4.4)	46 (4.4)	57 (4.4)
Retired	612 (25.9)	279 (26.4)	333 (25.5)
Long-term non-employed/stay-at-home	55 (2.3)	9 (0.9)	46 (3.5)
Volunteer work/parental leave/other leave	19 (0.8)	0 (0.0)	19 (1.5)
**Household income**			
< 1250 €	222 (9.4)	86 (8.2)	136 (10.5)
1250–2500 €	756 (32.1)	327 (31.0)	427 (32.9)
> 2500 €	1378 (58.5)	641 (60.8)	736 (56.7)

**Note**. M = mean, SD = standard deviation; *n* = 8 subjects did not report marital status; *n* = 3 unmarried subjects did not report relationship status: *n* = 2 subjects did not report education; *n* = 4 subjects did not report occupational status; *n* = 12 subjects did not report household income; *n* = 3 participants with diverse gender were included in the total sample but not shown separately; Percentages are calculated excluding missing values per variable

### Descriptive statistics and internal consistency

Item statistics are presented in Table 2. The highest mean item scores were observed for item 11 (“Negative emotions are harmful“; M = 4.59, SD = 1.69) and item 3 (“There is very little use for negative emotions”; M = 4.27, SD = 1.79), while the lowest scores were found for item 16 (“Positive emotions are harmful”, M = 1.89, SD = 1.39) and item 4 (“Positive emotions are very unhelpful to people”, M = 1.91, SD = 1.34). The items generally exhibit low to moderate skewness and largely unremarkable kurtosis, although some items (particularly items regarding the usefulness of positive emotions) show greater skewness and increased kurtosis, suggesting possible floor effects.

Corrected item-total correlations ranged from *r* = .36 to *r* = .68. Corrected item-subscale correlations ranged from *r* = .53 to *r* = .71 and were mostly higher than the corresponding item-total correlations. The EBQ subscales demonstrated acceptable to good internal consistency (α = 0.79-0.88; ω = 0.79-0.88). The unweighted raw-item sum score also showed high internal consistency (α = 0.88; ω_total = 0.91), with ω_total estimated from the correlated three-factor CFA model. Reliability and descriptive statistics for EBQ total and subscale scores are provided in the supplementary materials (Tables S1-S2; Figure S1).

**Table 2:** Item characteristics of the German EBQ

	Item	Subscale	M (SD)	Skew	Kurtosis	*r* ^a^	*r* ^b^
1	Once people are experiencing negative emotions, there is nothing they can do about modifying them	1	3.11 (1.66)	0.41	-0.73	0.63	0.63
2	People cannot control their positive emotions	2	3.15 (1.6)	0.29	-0.70	0.55	0.55
3	There is very little use for negative emotions	3	4.27 (1.79)	-0.23	-0.89	0.40	0.53
4	Positive emotions are very unhelpful to people	4	1.91 (1.34)	1.55	1.80	0.50	0.70
5	It doesn’t matter how hard people try, they cannot change their negative emotions	1	3.08 (1.55)	0.40	-0.52	0.68	0.71
6	People cannot learn techniques to effectively control their positive emotions	2	3.14 (1.65)	0.38	-0.63	0.62	0.60
7	People don’t need their negative emotions	3	3.89 (1.85)	-0.04	-1.00	0.46	0.59
8	There is very little use for positive emotions	4	2.2 (1.41)	1.10	0.37	0.51	0.70
9	People cannot control their negative emotions	1	3.39 (1.55)	0.16	-0.64	0.61	0.61
10	It doesn’t matter how hard people try, they cannot change their positive emotions	2	3.2 (1.56)	0.28	-0.61	0.62	0.64
11	Negative emotions are harmful	3	4.59 (1.69)	-0.50	-0.51	0.36	0.57
12	People don’t need their positive emotions	4	2.04 (1.52)	1.53	1.55	0.42	0.65
13	People cannot learn techniques to effectively control their negative emotions	1	3.03 (1.63)	0.44	-0.63	0.65	0.58
14	Once people are experiencing positive emotions, there is nothing they can do about modifying them	2	3.36 (1.57)	0.15	-0.63	0.58	0.58
15	The presence of negative emotions is a bad thing for people	3	4.25 (1.81)	-0.27	-0.92	0.43	0.68
16	Positive emotions are harmful	4	1.89 (1.39)	1.54	1.53	0.44	0.68

**Note**. Item wording corresponds to the original English version of the EBQ. The German translation administered in the present study is provided in the Supplementary Material. Subscale 1 = Negative Controllability, Subscale 2 = Positive Controllability, Subscale 3 = Negative Usefulness, Subscale 4 = Positive Usefulness; M = mean, SD = standard deviation; r^a^ = corrected item-total correlation (item with total score excluding the item); r^b^ = corrected item-subscale correlation (item with subscale score excluding the item); Item’s range 1–7

### Confirmatory factor analyses and model comparison

**Fig. 1:**
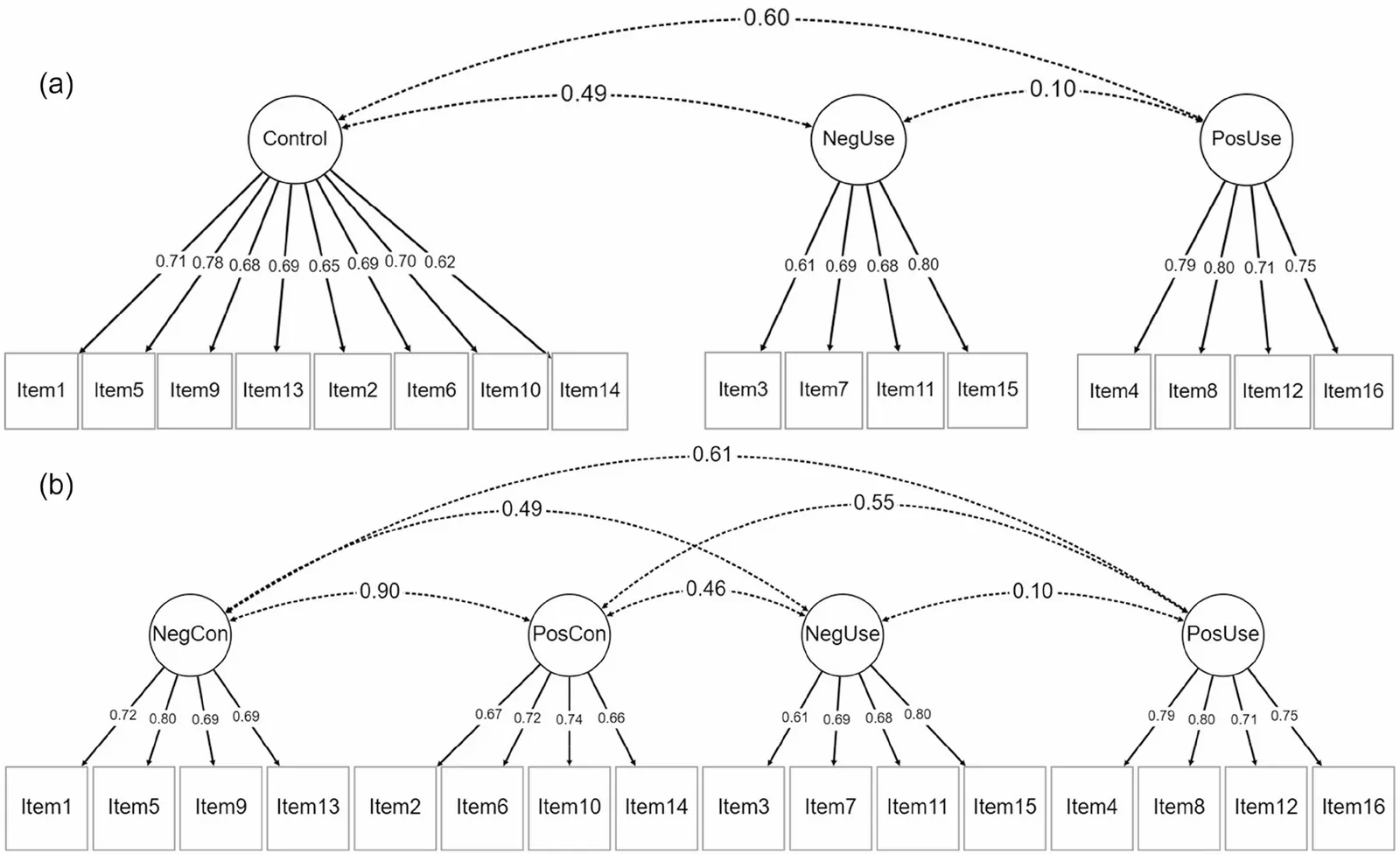
Confirmatory factor analysis of the three- and four-factor EBQ models with standardized factor loadings and latent factor correlations (robust MLR estimation); (a) Three-factor model with Controllability, Negative Usefulness, and Positive Usefulness; (b) Four-factor model with Negative Controllability, Positive Controllability, Negative Usefulness, and Positive Usefulness

Table 3 presents the global fit indices. The one-factor model showed poor fit with fit indices clearly below recommended thresholds [28]. The three-factor model, in which Positive and Negative Controllability were combined into a single factor, demonstrated acceptable fit (see Table 3), with a BIC difference of 116 points in favor of the four-factor solution. As depicted in Fig. 1, the latent correlation between the two controllability factors was high, *r* = .90, 95% CI [0.87, 0.93]; HTMT = 0.910. The ordinal sensitivity analysis yielded the same overall pattern of results: The three-factor model showed substantially better fit than the one-factor model, while the four-factor model again showed somewhat better fit than the three-factor model (Table S9). The latent correlation between Negative and Positive Controllability remained very high, *r* = .92, 95% CI [0.91, 0.93].

**Table 3:** Global fit indices of the 1-, 3-, and 4-factor models

	Scaled χ² (df)	CFI	TLI	RMSEA (90% CI)	SRMR	BIC
1-factor model	3514.4 (104)	0.680	0.630	0.144 [0.140, 0.148]	0.110	132,289
3-factor model	681.2 (101)	0.949	0.939	0.058 [0.054, 0.063]	0.039	128,043
4-factor model	586.4 (98)	0.957	0.947	0.054 [0.050, 0.058]	0.037	127,927

**Note**. χ² = chi-square; df = degrees of freedom; CFI = Comparative Fit Index; TLI = Tucker-Lewis Index; RMSEA = Root Mean Square Error of Approximation; CI = confidence interval; SRMR = Standardized Root Mean Square Residual; BIC = Bayesian Information Criterion. χ², CFI, TLI, and RMSEA are reported using robust corrections for MLR estimation. 90% CI refers to the robust RMSEA

Together, these findings indicate limited discriminant validity between Negative and Positive Controllability, whereas discriminant validity was acceptable for the remaining factors. Overall, the results support the factorial validity of the EBQ. However, the two controllability facets showed limited discriminant validity, raising questions about the practical utility of distinguishing them in scoring. In contrast, the three-factor model showed low to moderate latent factor intercorrelations (*r* = .10-0.60, see Fig. 1). The three-factor model was therefore retained for scoring purposes, based on parsimony and clearer discriminant validity. Detailed results for the three- and four-factor models, including latent factor correlations and factor loadings with standard errors, p values, and 95% confidence intervals, are provided in Tables S3-S6. Additional indices of discriminant validity, including AVE, HTMT, and the Fornell-Larcker criterion, are reported in Tables S2, S7 and S8.

### Sensitivity Analyses of Factor Structure and Score Interpretation

To examine whether the substantial overlap between Negative and Positive Controllability was attributable to their parallel item wording, we estimated a four-factor correlated-uniqueness model allowing residual covariances between the four corresponding negative/positive item pairs (see Table S10). Although model fit improved relative to the baseline four-factor model, as indicated by a Satorra-Bentler scaled chi-square difference test [29], Δχ²(4) = 38.73, *p* < .001, the latent correlation between Negative and Positive Controllability remained virtually unchanged (*r* = .902 vs. 0.898). Thus, the substantial overlap between the two controllability facets cannot be explained by parallel item wording, supporting their combination into a single Controllability factor for scoring purposes while acknowledging the better item-level fit of the four-factor model.

To evaluate the appropriateness of the EBQ total score considering its multidimensional structure, we fitted a bifactor model with one general factor loading on all 16 items and three orthogonal group factors (Controllability, Negative Usefulness, Positive Usefulness, Table S11). The model converged without Heywood cases or inadmissible estimates and showed good overall fit, robust CFI = 0.963, robust TLI = 0.949, robust RMSEA = 0.053, SRMR = 0.044, BIC = 127,904. Standardized general-factor loadings were generally higher for the Controllability items (0.62–0.79) than for the Positive Usefulness (0.39–0.49) and Negative Usefulness items (0.28–0.38), which in turn retained substantial loadings on their respective specific factors (Table S11, Panel B). Based on the standardized solution, the general factor showed an omega hierarchical of ωH = 0.792. The corresponding coefficient for the unweighted raw-item total score was highly similar (ωH = 0.788), indicating that the interpretation of the general factor was essentially unchanged for the score used in the descriptive reference values and regression analyses. Factor-score determinacy was high (0.949), indicating that the general factor was well represented by its factor-score estimates. Explained common variance (ECV = 0.603) and the percentage of uncontaminated correlations (PUC = 0.667) further indicated a substantial, although not exhaustive, general factor. Reise et al., [32] suggested that when PUC is below 0.80, the strength of the general factor should be considered jointly with ECV and ωH. In this situation, ECV values above 0.60 together with ωH values above 0.70 indicate that the multidimensionality may still be sufficiently limited to permit interpretation of a total score. The present values (PUC = 0.667, ECV = 0.603, ωH = 0.792) are consistent with this guideline, although ECV exceeds the suggested value only marginally. Thus, the findings support interpretation of the EBQ total score.

At the subscale level, Negative Usefulness (ωHS = 0.604) and Positive Usefulness (ωHS = 0.563) retained meaningful reliable variance beyond the general factor. In contrast, Controllability retained virtually no specific reliable variance (ωHS = 0.003; ECV = 0.036) and showed lower factor-score determinacy (0.641), indicating that its reliable variance was largely shared with the general factor. The corresponding ωHS coefficients for the unweighted raw-item subscale scores were virtually identical (Controllability = 0.003, Negative Usefulness = 0.604, Positive Usefulness = 0.562). This does not imply that the observed Controllability subscale is unreliable in the conventional sense (see Table S2), but that it provides little reliable variance beyond the general EBQ factor. As a robustness check, we additionally fitted a four-group-factor bifactor model separating Negative and Positive Controllability. This model showed virtually identical fit, robust CFI = 0.962, robust TLI = 0.949, robust RMSEA = 0.054, SRMR = 0.044, BIC = 127,908. Standardized general-factor loadings ranged from 0.281 to 0.791 (see Table S11, Panel D). General-factor indices were highly similar (ωH = 0.780, ECV = 0.580, PUC = 0.800, determinacy = 0.941), and both controllability-related group factors showed comparably low specific reliable variance (ωHS = 0.007 and 0.154, respectively), indicating that the limited incremental value of Controllability beyond the general factor is not an artifact of combining negative and positive controllability items. Taken together, these findings support retaining the EBQ total score, while qualifying its interpretation as a broad general index that more strongly reflects controllability beliefs than usefulness beliefs rather than as a strictly unidimensional measure.

### Descriptive reference values

**Fig. 2:**
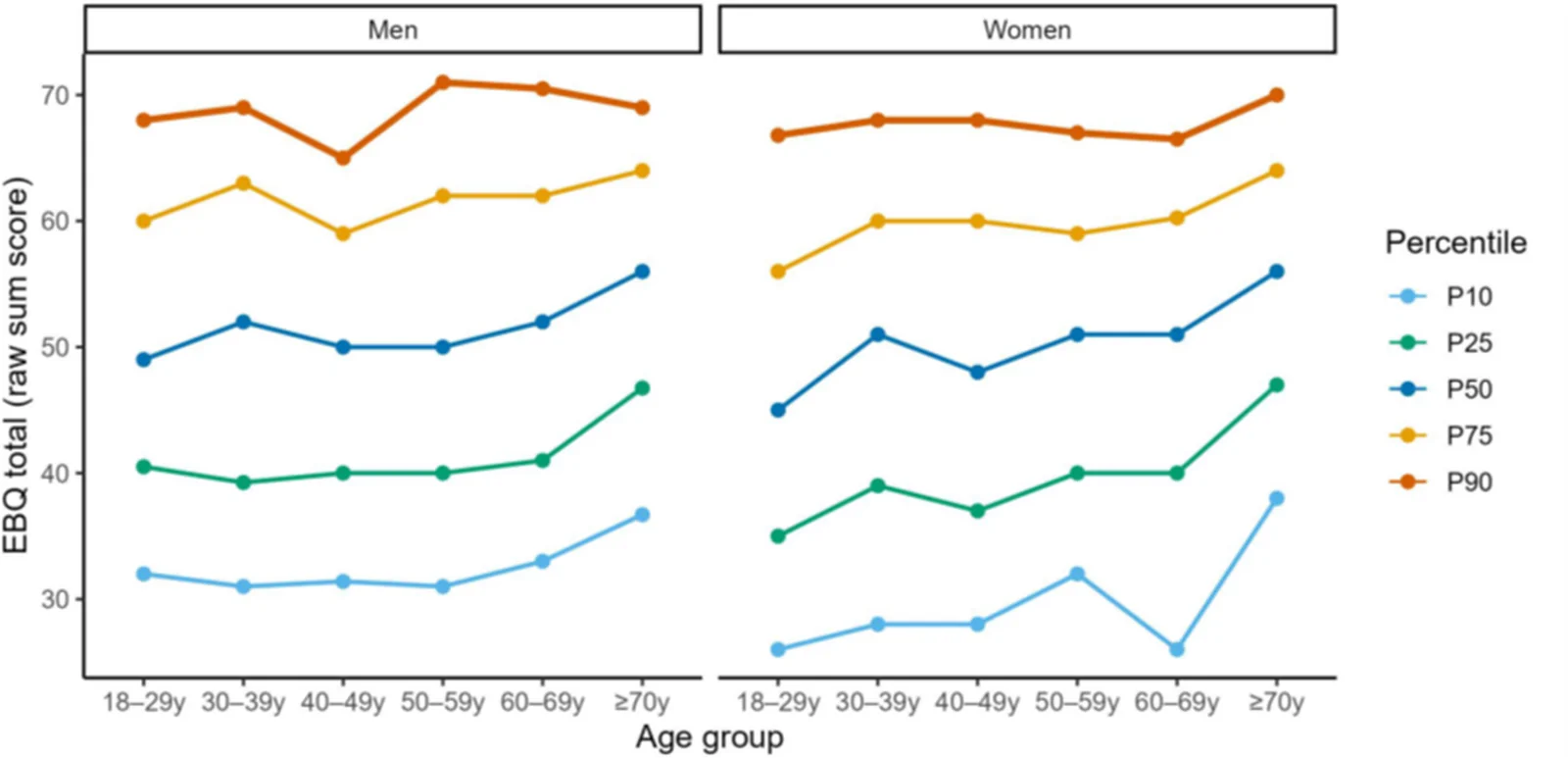
Percentile distributions of the EBQ total score by age group and gender

Descriptive percentile reference values for the EBQ total score stratified by gender and age group are presented in Table 4. Percentile values showed slight variation across age groups and between men and women (see also Fig. 2). Supplementary Tables S12-S14 provide corresponding descriptive percentile reference values for the EBQ subscales stratified by gender and age group. Overall, subgroup differences were small. For the Positive Usefulness subscale, several lower percentile ranks corresponded to the minimum attainable score, suggesting limited variability at the lower end of the distribution. Supplementary raw-score conversion tables providing empirical percentile ranks for each possible raw score of the EBQ total scale and the three subscales are presented in Tables S15-S16.

**Table 4:** Descriptive percentile reference values for the EBQ total score by gender and age group

EBQ percentile	Total		Men							Women					
	**18-93y**	**18-29y**		**30-39y**	**40-49y**	**50-59y**	**60-69y**	**≥ 70y**	**18-29y**		**30-39y**	**40-49y**	**50-59y**	**60-69y**	**≥ 70y**
1%	16	22		16	16	16	20	18	16		16	16	16	16	21
5%	25	26		25	24	26	28	30	20		24	23	24	19	31
10%	30	32		31	31	31	33	37	26		28	28	32	26	38
25%	40	40		39	40	40	41	47	35		39	37	40	40	47
50%	51	49		52	50	50	52	56	45		51	48	51	51	56
75%	61	60		63	59	62	62	64	56		60	60	59	60	64
90%	69	68		69	65	71	70	69	67		68	68	67	66	70
95%	74	77		74	76	76	75	74	73		72	74	72	72	77
99%	88	81		82	88	91	98	85	87		86	87	91	85	91

**Note**. y = years. Participants with diverse gender are included in the total sample but are not reported separately because of the small subgroup size (*n* = 3). Response scale: 1–7; theoretical raw sum score range: 16–112; 50% = median. The percentile values are descriptive reference values derived from the present population-based sample and are intended for research comparisons and preliminary clinical contextualization

### Measurement invariance

Measurement invariance was supported across gender and age up to the strict level, with only small changes in fit across increasingly restrictive models (Table 5). For education, the robust configural model showed CFI = 0.942, RMSEA = 0.061, and SRMR = 0.048, while the scalar model showed CFI = 0.933, RMSEA = 0.060, and SRMR = 0.054 (ΔCFI = − 0.0071, ΔRMSEA = 0.0006, ΔSRMR = 0.0019). Although all changes remained within the primary criteria (ΔCFI ≤ 0.010, ΔRMSEA ≤ 0.015; [35]; [34]), the decreases in CFI at the scalar and strict levels were somewhat larger under the more conservative sensitivity benchmark discussed by Chen [34] for small and unequal groups, including ΔCFI = − 0.0054 from scalar to strict. Together with the weaker group-specific fit in the two smallest education groups (Table S17), we interpret these findings as providing approximate support for scalar invariance across education groups under the continuous-item specification. Evidence for strict invariance was less conclusive. Local score-test diagnostics did not identify any loading, intercept, or residual-variance constraint that remained significant after correction for multiple testing (Table S18). Because scalar invariance is sufficient for latent mean comparisons [37], the approximate support for scalar invariance across education groups permits cautious interpretation of the substantive group comparisons reported in the manuscript. Strict invariance is not required for such comparisons.

**Table 5:** Measurement invariance across age, gender, and education for the three-factor EBQ model

	Model	Scaled χ²	df	CFI	RMSEA [90% CI]	SRMR	ΔCFI	ΔRMSEA	ΔSRMR
Age	Configural	1264.5	606	0.946	0.060 [0.055, 0.065]	0.049	-	-	-
	Metric	1365.8	671	0.944	0.058 [0.054, 0.063]	0.054	− 0.0019	− 0.0020	0.0050
	Scalar	1482.2	736	0.941	0.057 [0.053, 0.061]	0.055	− 0.0029	− 0.0012	0.0014
	Strict	1551.3	816	0.937	0.056 [0.052, 0.060]	0.057	− 0.0037	− 0.0012	0.0012
Gender	Configural	770.5	202	0.950	0.058 [0.053, 0.062]	0.041	-	-	-
	Metric	795.5	215	0.949	0.056 [0.052, 0.060]	0.042	− 0.0002	− 0.0016	0.0014
	Scalar	824.4	228	0.949	0.055 [0.051, 0.059]	0.042	− 0.0006	− 0.0013	0.0004
	Strict	807.1	244	0.950	0.053 [0.049, 0.057]	0.043	0.0009	− 0.0023	0.0001
Education	Configural	1326.9	606	0.942	0.061 [0.057, 0.066]	0.048	-	-	-
	Metric	1423.3	671	0.940	0.059 [0.055, 0.064]	0.053	− 0.0017	− 0.0022	0.0050
	Scalar	1593.3	736	0.933	0.060 [0.056, 0.064]	0.054	− 0.0071	0.0006	0.0019
	Strict	1672.4	816	0.928	0.059 [0.055, 0.063]	0.056	− 0.0054	− 0.0008	0.0015

**Note**. Group sizes: Age: < 30 years *n* = 356; 30–39 years *n* = 367; 40–49 years *n* = 399; 50–59 years *n* = 454; 60–69 years *n* = 452; ≥ 70 years *n* = 340 (*N* = 2368). Gender: men *n* = 1059; women *n* = 1306 (*N* = 2365, three participants with diverse gender were excluded). Education: basic school graduation *n* = 549; without graduation *n* = 60; middle maturity *n* = 989; technical school *n* = 99; A-level *n* = 357; university graduation *n* = 307 (*N* = 2361). Models were estimated using MLR with FIML. χ² denotes the scaled test statistic; df = degrees of freedom; CFI = Comparative Fit Index; RMSEA = Root Mean Square Error of Approximation; CI = confidence interval; SRMR = Standardized Root Mean Square Residual. Robust CFI and RMSEA values are reported, with 90% confidence intervals for RMSEA. Change values are reported to four decimal places because rounding to three would obscure comparisons with the 0.010 criterion. Criteria for evaluating invariance are described in the text

Results for the four-factor model were comparable (Tables S19-S20). Ordinal sensitivity analyses using WLSMV likewise supported threshold, loading, and intercept invariance across gender, age, and education, with only small changes in fit across successive invariance levels (|ΔCFI| ≤ 0.005; |ΔRMSEA| ≤ 0.013). These findings indicate that the substantive conclusions were robust to treating the EBQ items as ordered categorical indicators (Tables S21-S22).

### Impact of sociodemographic variables on EBQ scores

**Fig. 3:**
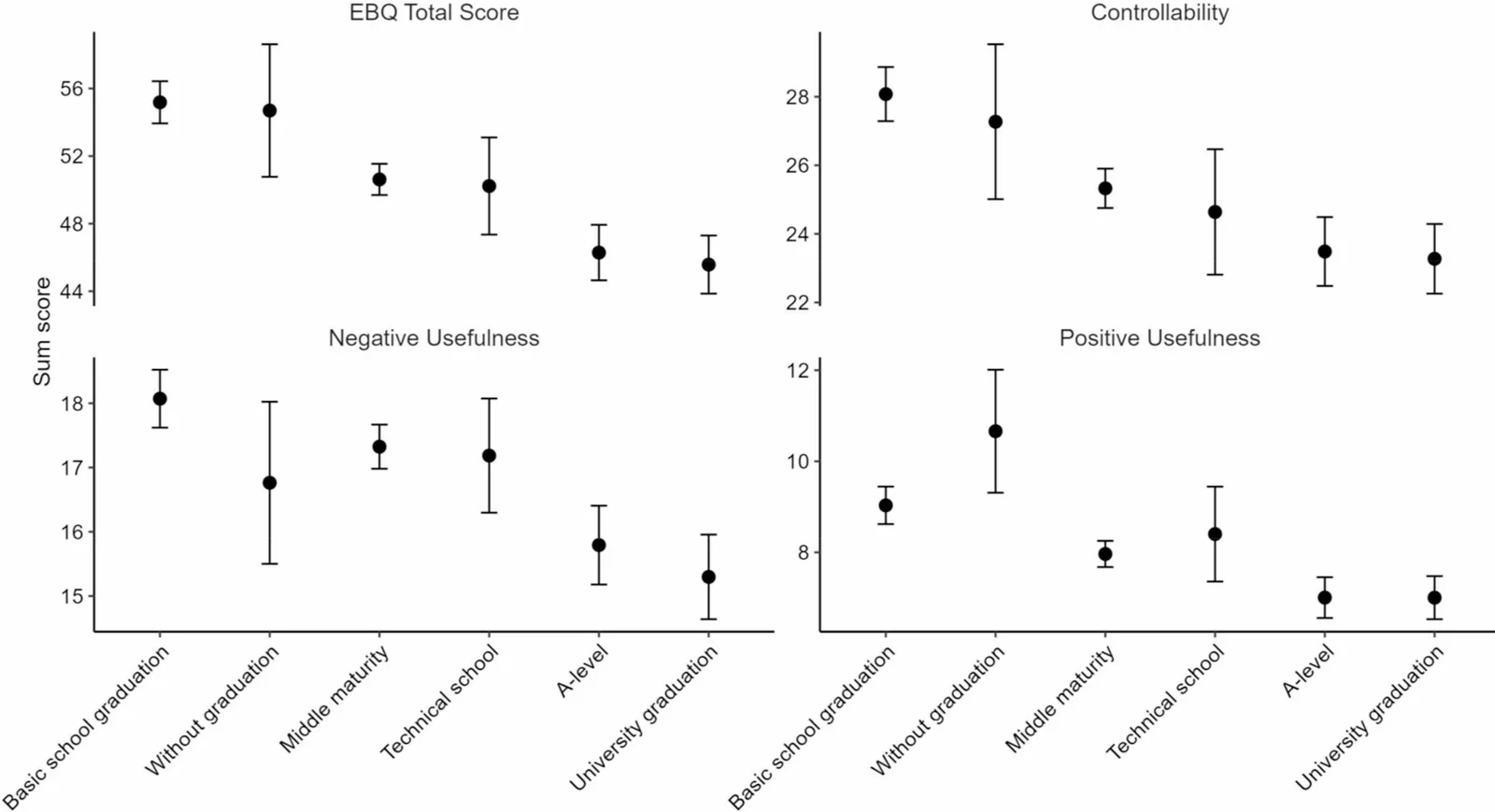
EBQ total score and subscale sum scores by educational level. Basic school graduation was used as a reference category in the regression. Higher EBQ scores reflect stronger maladaptive beliefs about emotions

Multiple linear regression analyses examined associations between sociodemographic variables and EBQ total and subscale scores. All four models were statistically significant (all *p* < .001), although explained variance was modest (R² = 0.037-0.060). For the EBQ total score, the model explained 6.0% of the variance, F(14, 2320) = 10.53, *p* < .001, R² = 0.060, adjusted R² = 0.054. Educational attainment showed the largest unique association, F(5, 2320) = 16.25, p_FDR < 0.001, partial R² = 0.034, ΔR² = 0.033 (Table 6 and S25). As depicted in Fig. 3, relative to basic school graduation, lower scores were observed for middle maturity (standardized coefficient = − 0.21, 95% CI [− 0.32, − 0.11]), A-level (− 0.49 [− 0.63, − 0.35]), and university graduation (− 0.56 [− 0.70, − 0.42]; all p_FDR < 0.001). The technical-school contrast did not remain significant after FDR correction (− 0.23 [− 0.45, − 0.02], p_FDR = 0.077), and the estimate for participants without graduation was imprecise (− 0.01 [− 0.28, 0.25]). Age showed a small positive association (0.08 [0.02, 0.13], p_FDR = 0.013), whereas women reported slightly lower scores than men (− 0.12 [− 0.20, − 0.04], p_FDR = 0.013). Although income and marital status were associated with EBQ total scores in bivariate analyses (see S27), their adjusted omnibus associations were not significant after FDR correction.

**Table 6:** Sociodemographic associations with EBQ total and subscale scores

Predictor	EBQ Total	Controllability	Negative Usefulness	Positive Usefulness
Age (centered)	0.08 [0.02, 0.13]	0.06 [0.01, 0.11]	0.05 [-0.00, 0.10]	0.07 [0.02, 0.13]
Gender: Women (ref. Men)	-0.12 [-0.20, -0.04]	-0.12 [-0.21, -0.04]	-0.05 [-0.13, 0.03]	-0.07 [-0.15, 0.01]
Education: Without graduation	-0.01 [-0.28, 0.25]	-0.08 [-0.35, 0.19]	-0.20 [-0.47, 0.06]	0.36 [0.09, 0.62]
Education: Middle maturity	-0.21 [-0.32, -0.11]	-0.21 [-0.32, -0.11]	-0.10 [-0.21, 0.01]	-0.15 [-0.26, -0.04]
Education: Technical school	-0.23 [-0.45, -0.02]	-0.28 [-0.50, -0.06]	-0.12 [-0.34, 0.09]	-0.05 [-0.27, 0.16]
Education: A-level	-0.49 [-0.63, -0.35]	-0.42 [-0.56, -0.28]	-0.34 [-0.48, -0.20]	-0.37 [-0.51, -0.23]
Education: University graduation	-0.56 [-0.70, -0.42]	-0.45 [-0.60, -0.31]	-0.46 [-0.60, -0.31]	-0.38 [-0.53, -0.24]
Income: €1250-<€2500	-0.11 [-0.26, 0.04]	-0.16 [-0.31, -0.01]	0.13 [-0.02, 0.28]	-0.18 [-0.33, -0.03]
Income: >€2500	-0.14 [-0.30, 0.01]	-0.20 [-0.36, -0.04]	0.06 [-0.09, 0.22]	-0.14 [-0.30, 0.01]
Marital status: Married (living without partner)	0.03 [-0.25, 0.30]	0.04 [-0.24, 0.32]	-0.13 [-0.41, 0.15]	0.16 [-0.12, 0.44]
Marital status: Unmarried (in a relationship)	0.05 [-0.10, 0.20]	0.09 [-0.06, 0.23]	-0.12 [-0.27, 0.03]	0.14 [-0.01, 0.28]
Marital status: Unmarried (not in a relationship)	-0.01 [-0.15, 0.12]	-0.02 [-0.15, 0.12]	-0.08 [-0.21, 0.06]	0.08 [-0.05, 0.22]
Marital status: Divorced	-0.08 [-0.21, 0.05]	-0.06 [-0.19, 0.07]	-0.05 [-0.18, 0.09]	-0.08 [-0.22, 0.05]
Marital status: Widowed	0.09 [-0.08, 0.26]	0.11 [-0.06, 0.28]	-0.10 [-0.27, 0.07]	0.20 [0.03, 0.37]
R²	0.060	0.044	0.037	0.045
Adjusted R²	0.054	0.039	0.031	0.039

Note. Cells show standardized coefficients with 95% confidence intervals. For age, coefficients are fully standardized (B × SDX / SDY); for categorical predictors, coefficients are outcome-standardized dummy-coded contrasts (B / SDY). Bold coefficients remained significant after Benjamini-Hochberg FDR correction across the 14 non-intercept coefficients within the respective outcome. Reference categories were men, basic school graduation, household income < €1250, and married and living with partner. Complete-case analysis; N = 2335

Subscale analyses showed a more differentiated pattern (Tables S23-S25). Educational attainment was the only predictor block significantly associated with all three subscales and showed the largest unique contribution in each model (ΔR² = 0.021-0.024, all p_FDR < 0.001). For Controllability, university graduation was associated with lower scores (− 0.45 [− 0.60, − 0.31]), as were female gender (− 0.12 [− 0.21, − 0.04]) and household income above €2500 (− 0.20 [− 0.36, − 0.04]). For Negative Usefulness, education was the only predictor block remaining significant after FDR correction, with the clearest contrasts for A-level (− 0.34 [− 0.48, − 0.20]) and university graduation (− 0.46 [− 0.60, − 0.31]). For Positive Usefulness, A-level (− 0.37 [− 0.51, − 0.23]) and university graduation (− 0.38 [− 0.53, − 0.24]) were associated with lower scores, whereas participants without a school-leaving qualification showed higher scores (0.36 [0.09, 0.62]); given the small size of this group (*n* = 59), this contrast should be interpreted cautiously. Marital status also showed a significant omnibus association with Positive Usefulness, F(5, 2320) = 2.98, p_FDR = 0.018, and age was positively associated with scores (0.07 [0.02, 0.13], p_FDR = 0.022). Some additional coefficient-level associations with income and marital status were observed but were less stable across sensitivity analyses (Tables S23-S25 and S29).

Regression diagnostics did not indicate substantial multicollinearity (adjusted GVIFs ≤ 1.31), and adding a quadratic age term did not improve model fit (all *p* ≥ .076, ΔR² ≤ 0.0013). Cook’s distances were small overall (maximum = 0.010). The Breusch-Pagan test indicated heteroskedasticity only for Negative Usefulness (*p* < .001). Sensitivity analyses using HC3 inference and FDR correction pooled across all four outcomes agreed with the primary analyses in 144 of 152 significance decisions. All eight discrepancies involved adjusted p values close to 0.05 (0.044-0.084; Table S29). Overall, educational attainment showed the largest and most consistent sociodemographic associations with EBQ scores, whereas associations with age, gender, income, and marital status were smaller and more outcome-specific. Given the modest explained variance (3.7%-6.0%), these associations should not be overinterpreted.

## Discussion

This study examined the psychometric properties of the German EBQ [3] in a representative sample of the German general population, including tests of measurement invariance across sociodemographic groups, descriptive reference values and sociodemographic correlates of emotion beliefs.

A key finding of the present study concerns the factor structure of the German EBQ. Confirmatory factor analyses showed better fit for the four-factor model than for the three-factor model on all indices examined (ΔCFI = 0.008, ΔTLI = 0.008, ΔRMSEA = 0.004, ΔBIC = 116). At the same time, the two controllability factors were very highly correlated, and neither the Fornell-Larcker criterion nor the HTMT criterion met recommended thresholds. These two lines of evidence point in different directions: the four-factor model captures item-level covariance more accurately, whereas the controllability facets it separates are barely distinguishable from one another in practice. This tension is not specific to the present sample. Comparably high intercorrelations between the controllability factors have been reported across linguistic and cultural contexts: *r* = .93 in a Polish [18], *r* = .97 in a U.S. general population sample [15], *r* = .87 in an Italian sample [17], and correlations between 0.94 and 0.95 across three samples including an Iranian one [16]. Previous validation studies retained and interpreted the four-factor model, generally without reporting formal tests of discriminant validity between Positive and Negative Controllability. Our sensitivity analyses further suggest that the high correlation is not primarily attributable to parallel item wording: freeing residual covariances between parallel-worded item pairs left the correlation between the two facets essentially unchanged. Complementary bifactor analyses further showed that Controllability retained virtually no reliable specific variance beyond the general factor. We therefore adopted the three-factor solution as a scoring decision grounded in parsimony and interpretability. The initial validation study by Becerra et al., [2] already favored a three-factor solution with a combined controllability factor and a general factor, based on conceptual considerations similar to those in the present study. Similarly, the Norwegian adaptation by Raanes et al., (2024) reported a high intercorrelation between the controllability factors (*r* = .90), favored the three-factor solution, and concluded that the EBQ “appears unable to effectively distinguish controllability beliefs by valence” (p. 290). Further support for the three-factor solution was recently reported in an Iranian clinical sample of adults undergoing psychotherapy (*N* = 385; [21]). Taken together, the consistently high intercorrelations across studies support the interpretation that beliefs about the controllability of positive and negative emotions, while statistically separable, overlap to a degree that limits their practical distinctness in the general population, and may largely reflect a broader, valence-independent belief in emotional controllability. Individuals who believe they can control positive emotions typically hold the same conviction regarding negative emotions and vice versa. Such a generalized sense of lacking emotional control has been linked to an increased risk of mental health difficulties across a range of psychopathological conditions, including depressive symptoms [11].

The present study provides population-based descriptive percentile reference values for the German EBQ. Such reference values allow individual scores to be contextualized against an appropriate reference population and may therefore facilitate the use of the EBQ in German-speaking research and preliminary clinical contextualization. From a clinical perspective, such reference values may be relevant because maladaptive beliefs about emotions may limit the extent to which individuals benefit from emotion regulation interventions. Although many psychotherapeutic approaches aim to strengthen adaptive regulation strategies, such strategies may be less readily adopted or applied when individuals view emotions as inherently uncontrollable or useless [9, 15]. Consistent with this, maladaptive emotion beliefs have been associated with psychological distress, maladaptive regulation strategies, and lower use of cognitive reappraisal [8, 11]. In a clinical sample, Mazidi et al., [21] identified six distinct emotion-belief profiles that differed meaningfully in their associations with emotion regulation difficulties and psychological distress. Notably, one profile was characterized by specific maladaptive beliefs about the uselessness of negative emotions despite largely adaptive beliefs about positive emotions, suggesting that clinically relevant emotion beliefs may differ not only in overall adaptiveness but also in their content and valence specificity. These findings indicate that interventions targeting maladaptive emotion beliefs may benefit from considering individuals’ specific belief profiles rather than conceptualizing maladaptive emotion beliefs as a uniform construct. Accordingly, future studies should examine whether the EBQ can support individualized intervention planning by identifying clinically meaningful patterns in beliefs about controllability, usefulness, or the adaptive functions of negative emotions. The reference values provided in the present study offer a basis for evaluating this potential clinical application, but such use requires further validation in clinical and prospective studies.

Establishing measurement invariance is essential to ensure that observed group differences reflect underlying differences in emotion beliefs rather than measurement artifacts [38]. The present study systematically examined measurement invariance of the German EBQ across age, gender, and educational level, with support up to the strict level for age and gender and approximate support at the scalar level for education. Previous studies have supported EBQ measurement invariance across gender and, in some cases, age or cultural groups (Larionow et al., 2023; [16, 17]). To date, Johnston et al., [15] appears to be the only study to have additionally examined invariance across educational groups. A German adaptation examined invariance only across gender [24], leaving age and education untested. This study’s results are particularly relevant in the light of the present findings on education. Educational attainment emerged as the most consistent sociodemographic correlate of EBQ scores, showing associations with the total score and all subscales after controlling for age, gender, income, and marital status (ΔR² = 0.021-0.033, p_FDR < 0.001). Although these effects were modest and the regression models explained only a small proportion of variance overall, their consistency suggests that educational attainment may nevertheless represent a relevant correlate of emotion beliefs. Higher educational attainment was associated with more adaptive emotion beliefs, a pattern not previously documented in the EBQ literature. The approximate scalar invariance observed across educational groups further supports interpreting these differences as substantive variation in emotion beliefs rather than as an artifact of differential measurement functioning.

Prior research has shown that lower SES is associated with greater emotional distress and may moderate the effectiveness of adaptive regulation strategies such as cognitive reappraisal [26]. One possible explanation is that educational attainment is associated with broader social, emotional, and language-based competencies in adulthood [39], which may support more differentiated representations of emotional experience. Emotional granularity - the ability to differentiate and label emotional states - has been linked to adaptive emotion regulation and psychological wellbeing [40, 41]. Individuals with higher educational attainment may therefore have greater resources for interpreting emotional experiences as understandable, manageable, and potentially useful. In line with Ford and Gross’s [1] model, such beliefs may constitute one pathway linking education to regulatory outcomes, consistent with evidence that lower educational attainment is associated with greater reliance on maladaptive strategies such as suppression and avoidance coping [42]. Notably, individuals with lower educational attainment are less likely to access mental health services and more likely to rely on pharmacological rather than psychological interventions, including antidepressants and sedatives [43]. This suggests that structural and psychological barriers to engaging with psychotherapy may be particularly relevant in this group. The present findings raise the possibility that less adaptive emotion beliefs constitute an additional belief-level barrier, by reducing the perceived usefulness, acceptability, or feasibility of engaging with emotion-focused psychological interventions and regulation strategies. Because emotion beliefs are thought to shape whether and how individuals engage in emotion regulation [1], education-sensitive interventions may benefit from targeting not only specific regulation strategies, but also beliefs about the controllability and usefulness of emotions. Such components could be integrated into psychoeducational or emotion regulation interventions using accessible language, concrete examples, and everyday emotion-related scenarios. Future research should examine whether education-sensitive adaptations enhance intervention efficacy and psychotherapy engagement among individuals with lower educational attainment, and whether changes in emotion beliefs mediate improvements in both engagement and emotion regulation outcomes across educational groups.

Biel et al., [3] noted a low factor loading for Item 16 (“It is harmful to feel positive emotions”; factor loading = 0.26) in their initial validation and recommended revising the item wording. In the present population-representative sample, however, the item showed no psychometric irregularities and did not compromise the scale. This finding is methodologically relevant, as the weak item performance reported by Biel et al., [3] may have been related to their small and demographically unbalanced convenience sample, which consisted predominantly of female and highly educated participants. The robust performance of Item 16 in the present study suggests that the item works adequately in representative samples and that revision of its wording does not appear necessary at this stage.

Several limitations should be noted. First, the cross-sectional design precludes causal conclusions. It remains unclear whether higher educational attainment contributes to more adaptive emotion beliefs or whether third variables, such as cognitive ability or stable personality traits, account for this association. Moreover, the regression models explained only a small proportion of variance in EBQ scores, indicating that sociodemographic characteristics capture only a limited part of individual differences in emotion beliefs. Second, as a self-report measure, the EBQ captures consciously held, trait-level emotion beliefs only. It does not assess implicit or situationally activated beliefs, which may be more closely linked to actual regulatory behavior [6]. Third, educational attainment serves as a broad proxy for the proposed mechanisms, such as emotional granularity or access to cognitive-reflective resources. Because these mechanisms were not directly assessed in the present study, the interpretation of the observed education effects remains speculative, and future studies should operationalize these constructs more explicitly to test the proposed pathways. Fourth, the near-zero specific reliable variance of the Controllability subscale (ωHS = 0.003) limits its interpretation as a distinct dimension. Although the subscale showed good internal consistency (Table S2), its reliable variance was almost entirely attributable to the general emotion-belief factor. Researchers using the EBQ should therefore exercise caution when interpreting Controllability as a distinct subscale and may wish to prioritize the Negative and Positive Usefulness subscales when subscale-specific interpretation is of interest.

The present findings point to several areas for further research. First, validation studies in clinical samples are necessary to derive clinically meaningful cut-off scores based on the current descriptive reference values and to examine the predictive validity of the EBQ for treatment response and relapse trajectories. Second, given the demands of clinical screening settings and large-scale population studies, developing an economical short-form version of the EBQ would be a sensible next step. Item response theory approaches could facilitate the selection of highly informative items while preserving measurement precision across the relevant range of emotion beliefs. Third, the range of language versions of the EBQ now available (including English, German, Polish, Turkish, Persian, Italian, and Norwegian) provides an important foundation for cross-cultural studies that can clarify whether the observed education-related associations are replicable across cultures. Finally, future longitudinal and intervention studies should investigate the extent to which changes in emotion beliefs precede and mediate changes in emotion regulation strategies over time.

## Conclusion

In conclusion, this study provides a comprehensive psychometric evaluation of the German version of the Emotion Beliefs Questionnaire in a large, population-representative adult sample. Extending prior work on the EBQ, the findings support a three-factor model comprising Controllability, Negative-Usefulness, and Positive-Usefulness as a parsimonious scoring solution. The study further provides strong evidence of measurement invariance across age and gender and approximate support for scalar invariance across educational groups, supporting meaningful group comparisons with some caution for educational level. In addition, the study provides the first population-based descriptive reference values for a German EBQ version, facilitating the interpretation of individual scores relative to the general adult population in research and preliminary clinical contextualization. Importantly, educational attainment emerged as the most consistent sociodemographic correlate of emotion beliefs, highlighting a potentially relevant sociocognitive factor that may contribute to inequalities in emotion regulation resources and mental health outcomes. Taken together, the findings support the EBQ as a psychometrically robust instrument for assessing emotion beliefs and underscore the relevance of these beliefs as a transdiagnostic and potentially modifiable construct. Further research should extend these findings in clinical, longitudinal, and cross-cultural contexts and examine how the EBQ can best be used for efficient and clinically informative assessment.

## Supplementary Information

Below is the link to the electronic supplementary material.

Supplementary Material 1

## Data Availability

The datasets used and analyzed during the current study are available from the corresponding author on reasonable request.

## Declarations

**Declaration of AI-assisted technologies**.

During the preparation of this work, the authors used ChatGPT (OpenAI) for technical assistance with debugging and refining R code used for statistical analyses as well as for language improvements. All code, analyses, statistical outputs and texts were independently reviewed and verified by the authors, who take full responsibility for the reported results.

## Abbreviations

EBQ: Emotion Beliefs Questionnaire
SES: Socio-economic Status
