# Supplementary material for: The German emotion beliefs questionnaire: factorial validity, measurement invariance, and descriptive reference values in a population-representative sample
Source: BMC Psychol. 2026 Oct 23;14:1629. doi: 10.1186/s40359-026-05837-z (PMC13753235; doi:10.1186/s40359-026-05837-z)
Supplement: Supplementary file 1 [file 40359_2026_5837_MOESM1_ESM.docx]

# Supplement

**1 EBQ Items**
List of German EBQ Items
S1. EBQ sum scores (M, SD) by age group and gender

**2 Descriptive Statistics**
S2. Reliability coefficients for the EBQ total score and factor scores and average variance extracted for the EBQ factors
Figure S1. EBQ total and subscale sum scores by age group and gender

**3 Detailed CFA Results**
S3. Latent factor correlations for the three-factor model
S4. Latent factor correlations for the four-factor model
S5. Standardized factor loadings of the three-factor EBQ model
S6. Standardized factor loadings of the four-factor EBQ model
S7. HTMT matrix for discriminant validity
S8. Fornell-Larcker matrix for discriminant validity
S9. Global fit indices of the ordinal 1-, 3-, and 4-factor models (WLSMV)
S10. Comparison of the baseline and correlated-uniqueness four-factor models
S11. Bifactor indices and standardized factor loadings for the three- and four-group-factor specifications

**4 Descriptive Reference Values**
S12. Descriptive reference values for the Controllability subscale by gender and age group
S13. Descriptive reference values for the Negative Usefulness subscale by gender and age group
S14. Descriptive reference values for the Positive Usefulness subscale by gender and age group
S15. Raw-score to percentile-rank conversion for the EBQ total sum score
S16. Raw-score to percentile-rank conversion for the EBQ subscales

**5 Measurement Invariance**
S17. Group-specific CFA fit (education)
S18. Local invariance diagnostics across education groups
S19. Fit indices of the measurement invariance (four-factor model)
S20. Changes in the fit indices across invariance levels (four-factor model)
S21. Fit indices of the ordinal measurement invariance models across age groups, gender, and education groups (three-factor model, WLSMV)
S22. Changes in fit indices across levels of ordinal measurement invariance (three-factor model, WLSMV)

**6 Regression**
S23. Model summaries for the EBQ total score and subscales
S24. Complete sociodemographic regression results (Panels A-D)
S25. Omnibus predictor-block tests and unique effect sizes
S26. Bivariate associations between age and EBQ outcomes
S27. Bivariate omnibus associations for categorical sociodemographic variables
S28. Regression diagnostics across EBQ outcomes
S29. Significance decisions differing between primary and sensitivity analyses

# 1 EBQ Items

Bitte lesen Sie aufmerksam jede der folgenden 16 Behauptungen durch und markieren Sie eine Zahl von 1-7, je nachdem, in welchem Maße diese Behauptung für Sie zutrifft.

| trifft überhaupt nicht zu |  |  | weder Zustimmung noch Ablehnung |  |  | trifft vollständig zu |
| --- | --- | --- | --- | --- | --- | --- |
| 1 | 2 | 3 | 4 | 5 | 6 | 7 |

1) Wenn Menschen erst einmal negative Gefühle erleben, können sie diese durch nichts verändern

2) Menschen können ihre positiven Gefühle nicht kontrollieren

3) Es gibt sehr wenig Nutzen von negativen Gefühlen

4) Positive Gefühle sind nutzlos für Menschen

5) Wie sehr sich die Menschen auch anstrengen, sie können ihre negativen Gefühle nicht verändern

6) Menschen können keine Techniken erlernen, um ihre positiven Gefühle wirksam zu kontrollieren

7) Menschen brauchen keine negativen Gefühle

8) Der Nutzen von positiven Gefühlen ist sehr gering

9) Menschen können ihre negativen Gefühle nicht kontrollieren

10) Wie sehr sich Menschen auch anstrengen, sie können ihre positiven Gefühle nicht verändern

11) Negative Gefühle sind gefährlich

12) Menschen brauchen ihre positiven Gefühle nicht

13) Menschen können keine Techniken erlernen, um ihre negativen Gefühle wirksam zu kontrollieren

14) Wenn Menschen erst einmal positive Gefühle erleben, können sie diese durch nichts verändern

15) Das Vorhandensein negativer Gefühle ist für Menschen eine schlechte Sache

16) Es ist schädlich, positive Gefühle zu empfinden

(German Translation, Biel et al., 2023)

| **Table S1. EBQ sum scores (M, SD) by age group and gender** | | | | | | | |
| --- | --- | --- | --- | --- | --- | --- | --- |
| **Group** | **N** | **EBQ Total Sum M (SD)** | **NegCon Sum M (SD)** | **PosCon Sum M (SD)** | **Con-trol Sum M (SD)** | **NegUse Sum M (SD)** | **PosUse Sum M (SD)** |
| **Total sample** | 2368 | 50.50 (15.33) | 12.60 (5.11) | 12.86 (4.98) | 25.46 (9.39) | 16.99 (5.57) | 8.05 (4.69) |
| <30 years | 356 | 47.90 (15.38) | 12.06 (4.95) | 12.15 (5.07) | 24.21 (9.33) | 16.13 (5.92) | 7.56 (4.53) |
| 30-39 years | 367 | 50.00 (15.42) | 12.40 (5.19) | 12.59 (5.12) | 24.99 (9.62) | 17.16 (5.80) | 7.84 (4.53) |
| 40-49 years | 399 | 49.27 (15.46) | 12.28 (5.23) | 12.59 (4.94) | 24.87 (9.38) | 16.73 (5.76) | 7.66 (4.67) |
| 50-59 years | 454 | 50.43 (15.28) | 12.55 (4.98) | 12.85 (4.82) | 25.40 (9.10) | 16.79 (5.32) | 8.24 (4.88) |
| 60-69 years | 452 | 50.76 (15.67) | 12.60 (5.22) | 13.04 (5.17) | 25.64 (9.69) | 17.10 (5.59) | 8.02 (4.58) |
| ≥70 years | 340 | 54.96 (13.69) | 13.86 (4.91) | 13.94 (4.58) | 27.79 (8.79) | 18.14 (4.81) | 9.02 (4.77) |
| **Men** | 1059 | 51.34 (15.19) | 12.93 (5.17) | 13.10 (4.95) | 26.03 (9.44) | 17.13 (5.53) | 8.18 (4.70) |
| <30 years | 163 | 49.91 (14.42) | 12.64 (4.92) | 12.92 (4.85) | 25.56 (8.98) | 16.74 (5.83) | 7.61 (4.36) |
| 30-39 years | 158 | 50.85 (15.69) | 12.94 (5.35) | 13.06 (5.29) | 25.99 (10.07) | 16.56 (5.58) | 8.30 (4.64) |
| 40-49 years | 163 | 49.65 (14.85) | 12.49 (5.22) | 12.79 (4.81) | 25.28 (9.35) | 16.74 (5.79) | 7.63 (4.62) |
| 50-59 years | 211 | 50.90 (15.88) | 12.81 (5.05) | 12.82 (4.92) | 25.63 (9.37) | 16.88 (5.39) | 8.39 (4.90) |
| 60-69 years | 216 | 52.24 (15.66) | 13.11 (5.31) | 13.26 (5.08) | 26.37 (9.66) | 17.57 (5.37) | 8.30 (4.92) |
| ≥70 years | 148 | 54.57 (13.77) | 13.65 (5.13) | 13.88 (4.66) | 27.53 (9.06) | 18.27 (5.16) | 8.78 (4.56) |
| **Women** | 1306 | 49.84 (15.42) | 12.34 (5.06) | 12.66 (5.00) | 25.00 (9.33) | 16.89 (5.61) | 7.95 (4.67) |
| <30 years | 193 | 46.19 (15.99) | 11.56 (4.95) | 11.50 (5.18) | 23.07 (9.49) | 15.61 (5.96) | 7.51 (4.69) |
| 30-39 years | 207 | 49.37 (15.27) | 11.97 (5.06) | 12.24 (4.98) | 24.21 (9.24) | 17.64 (5.96) | 7.52 (4.44) |
| 40-49 years | 235 | 49.06 (15.91) | 12.16 (5.25) | 12.48 (5.03) | 24.63 (9.42) | 16.74 (5.76) | 7.69 (4.71) |
| 50-59 years | 243 | 50.02 (14.77) | 12.32 (4.92) | 12.88 (4.74) | 25.21 (8.88) | 16.71 (5.26) | 8.10 (4.86) |
| 60-69 years | 236 | 49.40 (15.59) | 12.14 (5.10) | 12.84 (5.24) | 24.97 (9.70) | 16.66 (5.76) | 7.77 (4.25) |
| ≥70 years | 192 | 55.26 (13.65) | 14.02 (4.75) | 13.98 (4.53) | 28.00 (8.60) | 18.04 (4.53) | 9.21 (4.93) |
| **Note.** M = mean, SD = standard deviation. EBQ = Emotion Beliefs Questionnaire; NegCon = Negative Controllability; PosCon = Positive Controllability; NegUse = Negative Usefulness; PosUse = Positive Usefulness. Control represents the combined Controllability score (NegCon + PosCon), and Use represents the combined Usefulness score (NegUse + PosUse). Higher scores indicate stronger maladaptive beliefs regarding the uncontrollability and lack of usefulness of emotions. Participants identifying as a gender other than male or female were included in the total sample but are not presented separately due to the small subgroup size (N = 3). | | | | | | | |

# 2 Descriptive Statistics

| **Table S2. Reliability coefficients for the EBQ total score and factor scores and average variance extracted for the EBQ factors** | | | |
| --- | --- | --- | --- |
| **Factor** | **Omega (**ω) | **Cronbach’s Alpha** | **AVE** |
| Negative Controllability | .821 | .813 | .527 |
| Positive Controllability | .789 | .787 | .483 |
| Negative Usefulness | .794 | .785 | .489 |
| Positive Usefulness | .844 | .846 | .578 |
| Controllability | .879 | .879 | .477 |
| EBQ total score | .907 | .880 | / |
| **Note**. EBQ = Emotion Beliefs Questionnaire. Controllability represents the combined score of the three-factor model (Negative Controllability and Positive Controllability), whereas Negative Controllability and Positive Controllability are reported separately in line with the four-factor model. Omega coefficients for the factor scores reflect model-based composite reliability. For the EBQ total score, ω_total was estimated from the correlated three-factor CFA model and reflects reliable variance attributable to all three correlated factors. It should therefore not be interpreted as omega hierarchical or as evidence of a single general factor. Bifactor-based omega hierarchical and omega hierarchical subscale coefficients, together with the corresponding general- and specific-factor loadings, are reported in Table S11. | | | |

**Figure S1.** EBQ total and subscale sum scores by age group and gender.

# 3 Detailed CFA Results

| **Table S3. Latent Factor Correlations for the Three-Factor Model** | | | | | | | |
| --- | --- | --- | --- | --- | --- | --- | --- |
|  | |  | **r (unstd.)** | **SE** | **p** | **r (std.)** | **95% CI** |
| Controllability | Negative Usefulness | | 0.489 | 0.024 | < .001 | 0.489 | 0.443; 0.536 |
| Controllability | Positive Usefulness | | 0.599 | 0.019 | < .001 | 0.599 | 0.563; 0.636 |
| Negative Usefulness | Positive Usefulness | | 0.097 | 0.022 | < .001 | 0.097 | 0.054; 0.140 |
| **Note.** r (unstd.) = unstandardized latent factor correlation; r (std.) = standardized latent factor correlation; SE = standard error; CI = confidence interval. | | | | | | | |

| **Table S4. Latent Factor Correlations for the Four-Factor Model** | | | | | | | |
| --- | --- | --- | --- | --- | --- | --- | --- |
|  | |  | **r (unstd.)** | **SE** | **p** | **r (std.)** | **95% CI** |
| Negative Controllability | Positive Controllability | | 0.902 | 0.015 | < .001 | 0.902 | 0.872; 0.932 |
| Negative Controllability | Negative Usefulness | | 0.488 | 0.024 | < .001 | 0.488 | 0.441; 0.536 |
| Negative Controllability | Positive Usefulness | | 0.610 | 0.020 | < .001 | 0.610 | 0.571; 0.650 |
| Positive Controllability | Negative Usefulness | | 0.459 | 0.027 | < .001 | 0.459 | 0.407; 0.511 |
| Positive Controllability | Positive Usefulness | | 0.545 | 0.021 | < .001 | 0.545 | 0.505; 0.586 |
| Negative Usefulness | Positive Usefulness | | 0.097 | 0.022 | < .001 | 0.097 | 0.054; 0.141 |
| **Note.** r (unstd.) = unstandardized latent factor correlation; r (std.) = standardized latent factor correlation; SE = standard error; CI = confidence interval. | | | | | | | |

| **Table S5. Standardized factor loadings of the three-factor EBQ model** | | | | | | |
| --- | --- | --- | --- | --- | --- | --- |
| **Factor** | **Item** | **Coefficient** | **Standard error** | **z-value** | **p-value** | **95% CI** |
| Controllability | 1 | 0.710 | 0.015 | 48.942 | < .001 | 0.682; 0.738 |
| Controllability | 10 | 0.697 | 0.015 | 47.261 | < .001 | 0.669; 0.726 |
| Controllability | 13 | 0.689 | 0.015 | 44.920 | < .001 | 0.659; 0.719 |
| Controllability | 14 | 0.622 | 0.017 | 35.614 | < .001 | 0.588; 0.657 |
| Controllability | 2 | 0.650 | 0.016 | 41.387 | < .001 | 0.619; 0.681 |
| Controllability | 5 | 0.776 | 0.012 | 64.642 | < .001 | 0.752; 0.799 |
| Controllability | 6 | 0.691 | 0.015 | 45.254 | < .001 | 0.661; 0.721 |
| Controllability | 9 | 0.680 | 0.016 | 43.238 | < .001 | 0.650; 0.711 |
| Negative Usefulness | 11 | 0.675 | 0.017 | 39.707 | < .001 | 0.642; 0.709 |
| Negative Usefulness | 15 | 0.804 | 0.015 | 54.215 | < .001 | 0.775; 0.833 |
| Negative Usefulness | 3 | 0.610 | 0.020 | 30.512 | < .001 | 0.571; 0.649 |
| Negative Usefulness | 7 | 0.689 | 0.017 | 41.230 | < .001 | 0.656; 0.722 |
| Positive Usefulness | 12 | 0.709 | 0.020 | 34.597 | < .001 | 0.669; 0.749 |
| Positive Usefulness | 16 | 0.750 | 0.019 | 39.837 | < .001 | 0.713; 0.786 |
| Positive Usefulness | 4 | 0.789 | 0.016 | 48.391 | < .001 | 0.757; 0.821 |
| Positive Usefulness | 8 | 0.800 | 0.014 | 56.366 | < .001 | 0.772; 0.828 |

| **Table S6. Standardized factor loadings of the four-factor EBQ model** | | | | | | |
| --- | --- | --- | --- | --- | --- | --- |
| **Factor** | **Item** | **Coefficient** | **Standard error** | **z-value** | **p-value** | **95% CI** |
| Negative Controllability | 1 | .725 | 0.015 | 49.021 | < .001 | 0.696; 0.754 |
| Negative Controllability | 13 | .693 | 0.016 | 43.563 | < .001 | 0.662; 0.724 |
| Negative Controllability | 5 | .795 | 0.013 | 62.421 | < .001 | 0.770; 0.820 |
| Negative Controllability | 9 | .689 | 0.016 | 42.518 | < .001 | 0.657; 0.720 |
| Negative Usefulness | 11 | .675 | 0.017 | 39.640 | < .001 | 0.642; 0.708 |
| Negative Usefulness | 15 | .804 | 0.015 | 54.239 | < .001 | 0.774; 0.833 |
| Negative Usefulness | 3 | .610 | 0.020 | 30.533 | < .001 | 0.571; 0.649 |
| Negative Usefulness | 7 | .690 | 0.017 | 41.246 | < .001 | 0.657; 0.722 |
| Positive Controllability | 10 | .736 | 0.014 | 51.919 | < .001 | 0.708; 0.764 |
| Positive Controllability | 14 | .658 | 0.018 | 37.571 | < .001 | 0.623; 0.692 |
| Positive Controllability | 2 | .666 | 0.016 | 40.678 | < .001 | 0.634; 0.698 |
| Positive Controllability | 6 | .716 | 0.016 | 45.896 | < .001 | 0.685; 0.747 |
| Positive Usefulness | 12 | .709 | 0.020 | 34.584 | < .001 | 0.669; 0.749 |
| Positive Usefulness | 16 | .749 | 0.019 | 39.659 | < .001 | 0.712; 0.786 |
| Positive Usefulness | 4 | .789 | 0.016 | 48.601 | < .001 | 0.757; 0.821 |
| Positive Usefulness | 8 | .800 | 0.014 | 56.576 | < .001 | 0.773; 0.828 |

| **Table S7. HTMT matrix for discriminant validity** | | | | |
| --- | --- | --- | --- | --- |
|  | **Negative Controllability** | **Positive Controllability** | **Negative Usefulness** | **Positive Usefulness** |
| **Negative Controllability** | - |  |  |  |
| **Positive Controllability** | 0.910 | - |  |  |
| **Negative Usefulness** | 0.520 | 0.468 | - |  |
| **Positive Usefulness** | 0.602 | 0.539 | 0.068 | - |
| **Note.** Values represent HTMT ratios. Values below .85 indicate acceptable discriminant validity. | | | | |

| **Table S8. Fornell-Larcker matrix for discriminant validity** | | | | |
| --- | --- | --- | --- | --- |
| **Factor** | **Negative Controllability** | **Positive Controllability** | **Negative Usefulness** | **Positive Usefulness** |
| Negative Controllability | 0.726 | 0.902 | 0.488 | 0.610 |
| Positive Controllability | 0.902 | 0.695 | 0.459 | 0.545 |
| Negative Usefulness | 0.488 | 0.459 | 0.699 | 0.097 |
| Positive Usefulness | 0.610 | 0.545 | 0.097 | 0.760 |
| **Note.** Diagonal elements are the square roots of the average variance extracted. Off-diagonal elements are latent factor correlations from the four-factor model. Discriminant validity is supported when a diagonal element exceeds all correlations in its row and column. This criterion was not met for Negative and Positive Controllability (√AVE = .726 and .695 vs. r = .902). | | | | |

| **Table S9. Global fit indices of the ordinal 1-, 3-, and 4-factor models (WLSMV)** | | | | | |
| --- | --- | --- | --- | --- | --- |
|  | **Scaled χ² (df)** | **CFI** | **TLI** | **RMSEA (90% CI)** | **SRMR** |
| 1-factor model | 12409.8 (104) | .636 | .580 | .174 [0.170, 0.178] | .145 |
| 3-factor model | 1507.8 (101) | .936 | .925 | .074 [0.070, 0.078] | .045 |
| 4-factor model | 1399.1 (98) | .944 | .932 | .070 [0.066, 0.074] | .043 |
| **Note.** χ² = chi-square; df = degrees of freedom; CFI = Comparative Fit Index; TLI = Tucker-Lewis Index; RMSEA = Root Mean Square Error of Approximation; CI = confidence interval; SRMR = Standardized Root Mean Square Residual. Items were treated as ordered categorical indicators and models were estimated using WLSMV. The reported χ² is the WLSMV-adjusted test statistic. CFI, TLI, and RMSEA are robust fit indices. 90% CI refers to the robust RMSEA. | | | | | |

| **Table S10. Comparison of the baseline and correlated-uniqueness four-factor models.** | | | | | | |
| --- | --- | --- | --- | --- | --- | --- |
| **Model** | **CFI** | **TLI** | **RMSEA** | **SRMR** | **BIC** | **r (NegCont, PosCont) [95% CI]** |
| Four-factor model (baseline) | .957 | .947 | .054 | .037 | 127,927 | .902 [.872, .932] |
| Four-factor model (correlated uniqueness) | .961 | .950 | .053 | .037 | 127,889 | .898 [.868, .929] |
| **Note.** The correlated-uniqueness model allowed residual covariances between the four item pairs with parallel negative/positive wording (Items 1 and 14, 5 and 10, 9 and 2, and 13 and 6). Models were estimated using robust maximum likelihood (MLR). Robust CFI, TLI, and RMSEA values are reported. r(NegCont, PosCont) denotes the latent correlation between the Negative and Positive Controllability factors. | | | | | | |

| **Table S11. Bifactor indices and standardized factor loadings for the three- and four-group-factor specifications**  **Panel A. Bifactor indices (three-group-factor specification)** | |
| --- | --- |
| **Index** | **Value** |
| ECV (General factor) | .603 |
| ECV (Controllability) | .036 |
| ECV (Negative Usefulness) | .178 |
| ECV (Positive Usefulness) | .182 |
| Omega hierarchical (General factor, standardized solution) | .792 |
| Omega hierarchical subscale (Controllability, standardized solution) | .003 |
| Omega hierarchical subscale (Negative Usefulness, standardized solution) | .604 |
| Omega hierarchical subscale (Positive Usefulness, standardized solution) | .563 |
| Omega hierarchical (General factor, unweighted raw total score) | .788 |
| Omega hierarchical subscale (Controllability, unweighted raw score) | .003 |
| Omega hierarchical subscale (Negative Usefulness, unweighted raw score) | .604 |
| Omega hierarchical subscale (Positive Usefulness, unweighted raw score) | .562 |
| PUC | .667 |
| Factor score determinacy (General factor) | .949 |
| Factor score determinacy (Controllability) | .641 |
| Factor score determinacy (Negative Usefulness) | .876 |
| Factor score determinacy (Positive Usefulness) | .871 |

**Panel B. Standardized factor loadings (three-group-factor specification)**

| **Item** | **General factor loading** | **Specific factor** | **Specific factor loading** |
| --- | --- | --- | --- |
| Item 1 | .736 | Controllability | -.280 |
| Item 2 | .655 | Controllability | -.013 |
| Item 3 | .354 | Negative Usefulness | .474 |
| Item 4 | .480 | Positive Usefulness | .619 |
| Item 5 | .790 | Controllability | -.171 |
| Item 6 | .688 | Controllability | .184 |
| Item 7 | .375 | Negative Usefulness | .564 |
| Item 8 | .492 | Positive Usefulness | .621 |
| Item 9 | .677 | Controllability | -.024 |
| Item 10 | .700 | Controllability | .264 |
| Item 11 | .284 | Negative Usefulness | .615 |
| Item 12 | .388 | Positive Usefulness | .606 |
| Item 13 | .677 | Controllability | .070 |
| Item 14 | .621 | Controllability | .301 |
| Item 15 | .332 | Negative Usefulness | .770 |
| Item 16 | .411 | Positive Usefulness | .639 |

| **Panel C. Bifactor indices (four-group-factor specification)** | |
| --- | --- |
| **Index** | **Value** |
| ECV (General factor) | .580 |
| ECV (Negative Controllability) | .021 |
| ECV (Positive Controllability) | .049 |
| ECV (Negative Usefulness) | .175 |
| ECV (Positive Usefulness) | .175 |
| Omega hierarchical (General factor, standardized solution) | .780 |
| Omega hierarchical subscale (Negative Controllability, standardized solution) | .007 |
| Omega hierarchical subscale (Positive Controllability, standardized solution) | .154 |
| Omega hierarchical subscale (Negative Usefulness, standardized solution) | .602 |
| Omega hierarchical subscale (Positive Usefulness, standardized solution) | .550 |
| PUC | .800 |
| Factor score determinacy (General factor) | .941 |
| Factor score determinacy (Negative Controllability) | .557 |
| Factor score determinacy (Positive Controllability) | .627 |
| Factor score determinacy (Negative Usefulness) | .874 |
| Factor score determinacy (Positive Usefulness) | .864 |

**Panel D. Standardized factor loadings (four-group-factor specification)**

| **Item** | **General factor loading** | **Specific factor** | **Specific factor loading** |
| --- | --- | --- | --- |
| Item 1 | .718 | Negative Controllability | .258 |
| Item 2 | .620 | Positive Controllability | .188 |
| Item 3 | .357 | Negative Usefulness | .472 |
| Item 4 | .490 | Positive Usefulness | .611 |
| Item 5 | .791 | Negative Controllability | .218 |
| Item 6 | .666 | Positive Controllability | .224 |
| Item 7 | .382 | Negative Usefulness | .560 |
| Item 8 | .503 | Positive Usefulness | .612 |
| Item 9 | .680 | Negative Controllability | .056 |
| Item 10 | .642 | Positive Controllability | .432 |
| Item 11 | .281 | Negative Usefulness | .617 |
| Item 12 | .400 | Positive Usefulness | .598 |
| Item 13 | .734 | Negative Controllability | -.260 |
| Item 14 | .569 | Positive Controllability | .383 |
| Item 15 | .335 | Negative Usefulness | .768 |
| Item 16 | .417 | Positive Usefulness | .636 |
| **Note.** Standardized factor loadings are reported from the fully standardized solution. In both specifications, the general EBQ factor loaded on all 16 items and each item additionally loaded on one orthogonal specific factor. In the three-group-factor specification, Negative and Positive Controllability items formed a common Controllability factor. In the four-group-factor specification, they were modeled as separate specific factors. ECV = explained common variance; PUC = percentage of uncontaminated correlations. ECV, Omega hierarchical (ωH), and Omega hierarchical subscale (ωHS) coefficients labeled as “standardized solution” were calculated from the fully standardized solution. For the three-group-factor specification, additional Omega hierarchical, and Omega hierarchical subscale coefficients were calculated from the unstandardized bifactor solution for the unweighted raw-item sums used in the descriptive reference values and regression analyses. | | | |

# 4 Descriptive reference values

| **Table S12. Descriptive reference values for the Controllability subscale by gender and age group** | | | | | | | | | | | | | | | | | |
| --- | --- | --- | --- | --- | --- | --- | --- | --- | --- | --- | --- | --- | --- | --- | --- | --- | --- |
| **EBQ per-centile** | **Total** | | **Men** | | | | | | | **Women** | | | | | | | |
|  | **18-96y** | **18-29y** | | **30-39y** | **40-49y** | **50-59y** | **60-69y** | **≥70y** | **18-29y** | | **30-39y** | **40-49y** | **50-59y** | **60-69y** | **≥70y** |  |  |
| 1% | 8 | 8 | | 8 | 8 | 8 | 8 | 8 | 8 | | 8 | 8 | 8 | 8 | 8 |  |  |
| 5% | 9 | 9 | | 8 | 11 | 11 | 12 | 11 | 8 | | 9 | 10 | 10 | 8 | 13 |  |  |
| 10% | 13 | 15 | | 13 | 13 | 14 | 14 | 15 | 11 | | 12 | 12 | 13 | 11 | 16 |  |  |
| 25% | 19 | 19 | | 18 | 19 | 19 | 20 | 22 | 16 | | 18 | 18 | 19 | 18 | 23 |  |  |
| 50% | 26 | 26 | | 26 | 25 | 26 | 27 | 28 | 22 | | 24 | 24 | 26 | 26 | 29 |  |  |
| 75% | 32 | 32 | | 32 | 31 | 32 | 32 | 33 | 29 | | 31 | 32 | 32 | 32 | 33 |  |  |
| 90% | 37 | 37 | | 38 | 37 | 37 | 38 | 37 | 35 | | 35 | 36 | 35 | 36 | 38 |  |  |
| 95% | 41 | 41 | | 41 | 41 | 41 | 44 | 42 | 39 | | 38 | 41 | 38 | 39 | 43 |  |  |
| 99% | 49 | 48 | | 51 | 50 | 48 | 52 | 49 | 43 | | 49 | 47 | 49 | 54 | 48 |  |  |
| **Note.** y = years; Participants with diverse gender are included in the total sample but are not reported separately due to small subgroup size (n = 3); Response scale: 1-7; Theoretical raw sum score range for this scale: 8-56; 50% = Median. | | | | | | | | | | | | | | | | |  |

| **Table S13. Descriptive reference values for the Negative Usefulness subscale by gender and age group** | | | | | | | | | | | | | | | | | |
| --- | --- | --- | --- | --- | --- | --- | --- | --- | --- | --- | --- | --- | --- | --- | --- | --- | --- |
| **EBQ per-centile** | **Total** | | **Men** | | | | | | | **Women** | | | | | | | |
|  | **18-96y** | **18-29y** | | **30-39y** | **40-49y** | **50-59y** | **60-69y** | **≥70y** | **18-29y** | | **30-39y** | **40-49y** | **50-59y** | **60-69y** | **≥70y** |  |  |
| 1% | 4 | 6 | | 4 | 4 | 4 | 4 | 4 | 4 | | 4 | 4 | 4 | 4 | 4 |  |  |
| 5% | 7 | 7 | | 6 | 7 | 7 | 8 | 10 | 5 | | 6 | 5 | 8 | 6 | 10 |  |  |
| 10% | 9 | 9 | | 8 | 9 | 9 | 10 | 12 | 7 | | 9 | 8 | 10 | 9 | 13 |  |  |
| 25% | 14 | 12 | | 13 | 12 | 14 | 14 | 15 | 12 | | 14 | 14 | 13 | 13 | 16 |  |  |
| 50% | 17 | 17 | | 17 | 16 | 17 | 18 | 18 | 16 | | 19 | 18 | 17 | 17 | 18 |  |  |
| 75% | 21 | 21 | | 21 | 21 | 20 | 21 | 22 | 20 | | 22 | 21 | 20 | 20 | 21 |  |  |
| 90% | 24 | 24 | | 23 | 24 | 23 | 24 | 24 | 22 | | 25 | 24 | 23 | 24 | 23 |  |  |
| 95% | 26 | 25 | | 24 | 27 | 25 | 25 | 28 | 25 | | 26 | 24 | 26 | 26 | 25 |  |  |
| 99% | 28 | 28 | | 28 | 28 | 28 | 28 | 28 | 28 | | 28 | 28 | 28 | 28 | 27 |  |  |
| **Note.** y = years; Participants with diverse gender are included in the total sample but are not reported separately due to small subgroup size (n = 3); Response scale: 1-7; Theoretical raw sum score range for this scale: 4-28; 50% = Median. | | | | | | | | | | | | | | | | |  |

| **Table S14. Descriptive reference values for the Positive Usefulness subscale by gender and age group** | | | | | | | | | | | | | | |
| --- | --- | --- | --- | --- | --- | --- | --- | --- | --- | --- | --- | --- | --- | --- |
| **EBQ per-centile** | **Total** | **Men** | | | | | | | **Women** | | | | | |
|  | **18-96y** | **18-29y** | **30-39y** | **40-49y** | **50-59y** | **60-69y** | **≥70y** | **18-29y** | | **30-39y** | **40-49y** | **50-59y** | **60-69y** | **≥70y** |
| 1% | 4 | 4 | 4 | 4 | 4 | 4 | 4 | 4 | | 4 | 4 | 4 | 4 | 4 |
| 5% | 4 | 4 | 4 | 4 | 4 | 4 | 4 | 4 | | 4 | 4 | 4 | 4 | 4 |
| 10% | 4 | 4 | 4 | 4 | 4 | 4 | 4 | 4 | | 4 | 4 | 4 | 4 | 4 |
| 25% | 4 | 4 | 4 | 4 | 4 | 4 | 4 | 4 | | 4 | 4 | 4 | 4 | 5 |
| 50% | 6 | 6 | 7 | 6 | 7 | 7 | 8 | 5 | | 5 | 5 | 6 | 6 | 7 |
| 75% | 11 | 10 | 11 | 9 | 11 | 11 | 12 | 10 | | 10 | 10 | 10 | 10 | 13 |
| 90% | 16 | 15 | 16 | 16 | 16 | 16 | 16 | 15 | | 15 | 16 | 16 | 15 | 16 |
| 95% | 17 | 16 | 17 | 17 | 18 | 17 | 17 | 17 | | 17 | 18 | 18 | 16 | 17 |
| 99% | 21 | 20 | 20 | 19 | 23 | 24 | 19 | 21 | | 19 | 20 | 23 | 18 | 20 |
| **Note.** y = years; Participants with diverse gender are included in the total sample but are not reported separately due to small subgroup size (n = 3); Response scale: 1-7; Theoretical raw sum score range for this scale: 4-28; 50% = Median. | | | | | | | | | | | | | | |

| **Table S15. Raw-score to percentile-rank conversion for the EBQ total sum score** | | | | | | | |
| --- | --- | --- | --- | --- | --- | --- | --- |
| **Raw score** | **PR** | **Raw score** | **PR** | **Raw score** | **PR** | **Raw score** | **PR** |
| 16 | 0.9 | 41 | 27.4 | 66 | 86.1 | 91 | 99.4 |
| 17 | 1.9 | 42 | 29.6 | 67 | 87.5 | 92 | 99.5 |
| 18 | 2.1 | 43 | 31.9 | 68 | 89.1 | 93 | 99.6 |
| 19 | 2.3 | 44 | 34.2 | 69 | 90.4 | 94 | 99.7 |
| 20 | 2.8 | 45 | 36.3 | 70 | 91.4 | 95 | 99.8 |
| 21 | 3.2 | 46 | 38.7 | 71 | 92.4 | 96 | 99.8 |
| 22 | 3.6 | 47 | 41.1 | 72 | 93.1 | 97 | 99.8 |
| 23 | 4.1 | 48 | 43.5 | 73 | 93.9 | 98 | 99.8 |
| 24 | 4.7 | 49 | 46.0 | 74 | 94.7 | 99 | 99.8 |
| 25 | 5.4 | 50 | 48.4 | 75 | 95.4 | 100 | 99.9 |
| 26 | 6.3 | 51 | 50.4 | 76 | 95.8 | 101 | 99.9 |
| 27 | 7.1 | 52 | 52.1 | 77 | 96.2 | 102 | 99.9 |
| 28 | 7.9 | 53 | 54.5 | 78 | 96.6 | 103 | 99.9 |
| 29 | 8.8 | 54 | 57.1 | 79 | 97.0 | 104 | 100.0 |
| 30 | 9.7 | 55 | 59.5 | 80 | 97.1 | 105 | 100.0 |
| 31 | 10.6 | 56 | 61.6 | 81 | 97.4 | 106 | 100.0 |
| 32 | 11.8 | 57 | 63.9 | 82 | 97.8 | 107 | 100.0 |
| 33 | 13.2 | 58 | 66.4 | 83 | 98.1 | 108 | 100.0 |
| 34 | 14.5 | 59 | 69.1 | 84 | 98.3 | 109 | 100.0 |
| 35 | 16.0 | 60 | 71.6 | 85 | 98.4 | 110 | 100.0 |
| 36 | 17.8 | 61 | 74.1 | 86 | 98.6 | 111 | 100.0 |
| 37 | 19.5 | 62 | 76.6 | 87 | 98.8 | 112 | 100.0 |
| 38 | 21.1 | 63 | 78.7 | 88 | 99.0 |  |  |
| 39 | 22.7 | 64 | 81.5 | 89 | 99.2 |  |  |
| 40 | 24.9 | 65 | 84.5 | 90 | 99.3 |  |  |
| **Note.** PR = percentile rank, calculated as the percentage of participants scoring below the respective raw score plus one half of the percentage obtaining that score. Values are based on the total population-based sample (N = 2,368) and are not differentiated by gender or age group. The theoretical score range is 16-112. Higher scores indicate stronger maladaptive emotion beliefs. These values are descriptive reference values and should not be interpreted as validated clinical norms or diagnostic cut-offs. | | | | | | | |

| **Table S16.** **Raw-score to percentile-rank conversion for the EBQ subscales** | | | | | | | |
| --- | --- | --- | --- | --- | --- | --- | --- |
| **Controllability** | | | | **Negative Usefulness** | | **Positive Usefulness** | |
| **Raw score** | **PR** | **Raw score** | **PR** | **Raw score** | **PR** | **Raw score** | **PR** |
| 8 | 2.0 | 33 | 81.0 | 4 | 1.2 | 4 | 16.7 |
| 9 | 4.6 | 34 | 84.0 | 5 | 2.8 | 5 | 39.1 |
| 10 | 5.6 | 35 | 86.5 | 6 | 3.9 | 6 | 48.2 |
| 11 | 6.9 | 36 | 88.7 | 7 | 5.6 | 7 | 54.8 |
| 12 | 8.6 | 37 | 90.6 | 8 | 7.7 | 8 | 61.6 |
| 13 | 10.5 | 38 | 92.2 | 9 | 10.2 | 9 | 67.5 |
| 14 | 12.5 | 39 | 93.5 | 10 | 12.8 | 10 | 72.2 |
| 15 | 14.5 | 40 | 94.5 | 11 | 15.4 | 11 | 76.5 |
| 16 | 17.6 | 41 | 95.2 | 12 | 18.6 | 12 | 79.9 |
| 17 | 20.7 | 42 | 95.9 | 13 | 22.7 | 13 | 82.4 |
| 18 | 23.1 | 43 | 96.4 | 14 | 27.4 | 14 | 84.7 |
| 19 | 25.9 | 44 | 97.0 | 15 | 33.2 | 15 | 87.2 |
| 20 | 29.4 | 45 | 97.4 | 16 | 40.6 | 16 | 90.8 |
| 21 | 33.2 | 46 | 97.7 | 17 | 47.6 | 17 | 94.4 |
| 22 | 36.5 | 47 | 98.1 | 18 | 53.9 | 18 | 96.2 |
| 23 | 40.1 | 48 | 98.5 | 19 | 61.0 | 19 | 97.5 |
| 24 | 43.9 | 49 | 98.9 | 20 | 68.6 | 20 | 98.6 |
| 25 | 47.3 | 50 | 99.2 | 21 | 75.5 | 21 | 99.0 |
| 26 | 51.1 | 51 | 99.3 | 22 | 81.2 | 22 | 99.3 |
| 27 | 54.8 | 52 | 99.4 | 23 | 86.2 | 23 | 99.4 |
| 28 | 58.2 | 53 | 99.4 | 24 | 90.4 | 24 | 99.6 |
| 29 | 62.1 | 54 | 99.5 | 25 | 93.5 | 25 | 99.8 |
| 30 | 66.2 | 55 | 99.5 | 26 | 95.5 | 26 | 99.9 |
| 31 | 70.4 | 56 | 99.8 | 27 | 96.8 | 27 | 99.9 |
| 32 | 76.0 |  |  | 28 | 98.7 | 28 | 100.0 |
| **Note.** PR = percentile rank, calculated as the percentage of participants scoring below the respective raw score plus one half of the percentage obtaining that score. Values are based on the total population-based sample (N = 2368) and are not differentiated by gender or age group. Higher scores indicate stronger maladaptive emotion beliefs. | | | | | | | |

# 5 Measurement Invariance

| **Table S17. Group-specific CFA fit (education)** | | | | | | | |
| --- | --- | --- | --- | --- | --- | --- | --- |
| **Group** | **n** | **Scaled χ²** | **df** | **CFI** | **TLI** | **RMSEA** | **SRMR** |
| Without graduation | 60 | 168.1 | 101 | 0.816 | 0.782 | 0.105 [0.072, 0.136] | 0.100 |
| Basic school graduation | 549 | 205.9 | 101 | 0.959 | 0.952 | 0.052 [0.042, 0.062] | 0.042 |
| Middle maturity | 989 | 383.3 | 101 | 0.937 | 0.925 | 0.063 [0.056, 0.070] | 0.043 |
| Technical school | 99 | 159.4 | 101 | 0.908 | 0.891 | 0.078 [0.053, 0.102] | 0.090 |
| A-level | 357 | 190.9 | 101 | 0.955 | 0.947 | 0.056 [0.044, 0.068] | 0.046 |
| University graduation | 307 | 190.2 | 101 | 0.944 | 0.934 | 0.060 [0.047, 0.073] | 0.049 |
| Note. Each education group was fitted separately using MLR with FIML. χ² denotes the scaled test statistic; CFI, TLI, and RMSEA are the robust variants. All solutions converged and were admissible. | | | | | | | |

**Table S18. Local invariance diagnostics across education groups**

**Panel A. Loading invariance (metric model)**

| **Type** | **Item** | **Comparison** | **Scaled χ²** | **df** | **p** | **p_FDR** |
| --- | --- | --- | --- | --- | --- | --- |
| Loading | 14 | Basic school graduation vs. Technical school | 6.07 | 1 | .014 | .645 |
| Loading | 2 | Basic school graduation vs. Technical school | 5.08 | 1 | .024 | .645 |
| Loading | 5 | Basic school graduation vs. Without graduation | 4.66 | 1 | .031 | .645 |
| Loading | 15 | Basic school graduation vs. Technical school | 4.23 | 1 | .040 | .645 |
| Loading | 5 | Basic school graduation vs. Middle maturity | 3.06 | 1 | .080 | .815 |
| Loading | 6 | Basic school graduation vs. Without graduation | 3.03 | 1 | .082 | .815 |
| Loading | 12 | Basic school graduation vs. Technical school | 2.61 | 1 | .106 | .815 |
| Loading | 2 | Basic school graduation vs. Middle maturity | 2.51 | 1 | .113 | .815 |
| Loading | 16 | Basic school graduation vs. Technical school | 2.38 | 1 | .123 | .815 |
| Loading | 6 | Basic school graduation vs. University graduation | 1.95 | 1 | .162 | .815 |

**Panel B. Intercept invariance (scalar model)**

| **Type** | **Item** | **Comparison** | **Scaled χ²** | **df** | **p** | **p_FDR** |
| --- | --- | --- | --- | --- | --- | --- |
| Intercept | 10 | Basic school graduation vs. Without graduation | 10.18 | 1 | .001 | .102 |
| Intercept | 7 | Basic school graduation vs. University graduation | 7.88 | 1 | .005 | .102 |
| Intercept | 3 | Basic school graduation vs. Technical school | 7.60 | 1 | .006 | .102 |
| Intercept | 1 | Basic school graduation vs. A-level | 7.59 | 1 | .006 | .102 |
| Intercept | 13 | Basic school graduation vs. A-level | 7.43 | 1 | .006 | .102 |
| Intercept | 14 | Basic school graduation vs. University graduation | 6.94 | 1 | .008 | .112 |
| Intercept | 14 | Basic school graduation vs. Technical school | 6.24 | 1 | .012 | .142 |
| Intercept | 9 | Basic school graduation vs. Middle maturity | 6.02 | 1 | .014 | .142 |
| Intercept | 13 | Basic school graduation vs. Technical school | 5.57 | 1 | .018 | .153 |
| Intercept | 3 | Basic school graduation vs. Middle maturity | 5.24 | 1 | .022 | .153 |

**Panel C. Residual variance invariance (strict model)**

| **Type** | **Item** | **Comparison** | **Scaled χ²** | **df** | **p** | **p_FDR** |
| --- | --- | --- | --- | --- | --- | --- |
| Residual variance | 3 | Basic school graduation vs. Technical school | 6.96 | 1 | .008 | .295 |
| Residual variance | 8 | Basic school graduation vs. A-level | 6.49 | 1 | .011 | .295 |
| Residual variance | 4 | Basic school graduation vs. Technical school | 6.19 | 1 | .013 | .295 |
| Residual variance | 14 | Basic school graduation vs. Technical school | 5.32 | 1 | .021 | .295 |
| Residual variance | 16 | Basic school graduation vs. A-level | 5.30 | 1 | .021 | .295 |
| Residual variance | 11 | Basic school graduation vs. University graduation | 5.24 | 1 | .022 | .295 |
| Residual variance | 1 | Basic school graduation vs. University graduation | 3.52 | 1 | .061 | .555 |
| Residual variance | 4 | Basic school graduation vs. A-level | 3.34 | 1 | .068 | .555 |
| Residual variance | 16 | Basic school graduation vs. Without graduation | 3.30 | 1 | .069 | .555 |
| Residual variance | 5 | Basic school graduation vs. Without graduation | 3.28 | 1 | .070 | .555 |
| **Note.** Univariate scaled score tests were used to examine across-group equality constraints. Panel A presents tests of factor loadings in the metric model, Panel B tests of item intercepts in the scalar model, and Panel C tests of residual variances in the strict model. All comparisons use the basic school graduation group as the reference category. pFDR denotes Benjamini-Hochberg-adjusted p values, calculated separately within each panel. For each panel, the 10 largest scaled score tests are shown. No loading, intercept, or residual-variance constraint remained significant after FDR correction. | | | | | | |

| **Table S19. Fit indices of the measurement invariance (four-factor model)** | | | | |
| --- | --- | --- | --- | --- |
| **Variable** | **Model** | **CFI** | **RMSEA** | **SRMR** |
| Age group | Configural | .954 | .056 | .047 |
|  | Metric (Loadings) | .952 | .055 | .052 |
|  | Scalar (Loadings + Intercepts) | .949 | .054 | .053 |
|  | Strict (incl. Residuals) | .945 | .053 | .055 |
| Gender | Configural | .958 | .054 | .039 |
|  | Metric (Loadings) | .958 | .052 | .040 |
|  | Scalar (Loadings + Intercepts) | .957 | .051 | .040 |
|  | Strict (incl. Residuals) | .958 | .049 | .040 |
| Education | Configural | .951 | .057 | .046 |
|  | Metric (Loadings) | .950 | .055 | .050 |
|  | Scalar (Loadings + Intercepts) | .944 | .056 | .052 |
|  | Strict (incl. Residuals) | .937 | .056 | .053 |
| **Note.** CFI = Comparative Fit Index; RMSEA = Root Mean Square Error of Approximation; SRMR = Standardized Root Mean Square Residual. Robust CFI and RMSEA values are reported due to the use of the MLR estimator. | | | | |

| **Table S20. Changes in the fit indices across invariance levels (four-factor model)** | | | | |  |
| --- | --- | --- | --- | --- | --- |
|  |  | **ΔCFI** | **ΔRMSEA** | **ΔSRMR** | |
| Age group | Configural → Metric | -.0017 | -.0017 | .0048 | |
|  | Metric → Scalar | -.0032 | -.0007 | .0014 | |
|  | Scalar → Strict | -.0041 | -.0008 | .0013 | |
| Gender | Configural → Metric | -.0002 | -.0014 | .0014 | |
|  | Metric → Scalar | -.0006 | -.0011 | .0004 | |
|  | Scalar → Strict | .0009 | -.0023 | .0001 | |
| Education | Configural → Metric | -.0017 | -.0018 | .0046 | |
|  | Metric → Scalar | -.0060 | .0007 | .0017 | |
|  | Scalar → Strict | -.0061 | -.0001 | .0015 | |
| **Note.** Group sizes: Age: < 30 years n = 356; 30-39 years n = 367; 40-49 years n = 399; 50-59 years n = 454; 60-69 years n = 452; ≥ 70 years n = 340 (N = 2368). Gender: men n = 1059; women n = 1306 (N = 2365; three participants with diverse gender were excluded). Education: basic school graduation n = 549; without graduation n = 60; middle maturity n = 989; technical school n = 99; A-level n = 357; university graduation n = 307 (N = 2361). Models were estimated using MLR and robust CFI and RMSEA values are reported. Changes in fit were calculated from unrounded values. As primary criteria, invariance was considered supported when ΔCFI did not exceed .010 and ΔRMSEA did not exceed .015 (Cheung & Rensvold, 2002; Chen, 2007). Given the highly unequal education-group sizes and the small size of two groups, more stringent change-in-fit criteria discussed by Chen (2007) were additionally considered as a conservative sensitivity benchmark. Under this sensitivity analysis, the education results were less unequivocal at the scalar and strict levels. Education invariance is therefore interpreted as providing approximate rather than unqualified support at the scalar level. | | | | | |

| **Table S21. Fit indices of the ordinal measurement invariance models across age groups, gender, and education groups (three-factor model, WLSMV)** | | | | |
| --- | --- | --- | --- | --- |
| **Variable** | **Model** | **CFI** | **RMSEA** | **SRMR** |
| Age group | Configural | .962 | .081 | .056 |
|  | Threshold invariance | .959 | .068 | .056 |
|  | Threshold + loading | .963 | .063 | .057 |
|  | Threshold + loading + intercept | .965 | .059 | .057 |
| Gender | Configural | .964 | .077 | .048 |
|  | Threshold invariance | .962 | .068 | .048 |
|  | Threshold + loading | .966 | .064 | .048 |
|  | Threshold + loading + intercept | .968 | .060 | .048 |
| Education | Configural | .965 | .076 | .055 |
|  | Threshold invariance | .960 | .067 | .055 |
|  | Threshold + loading | .964 | .061 | .056 |
|  | Threshold + loading + intercept | .965 | .058 | .056 |
| **Note.** CFI = Comparative Fit Index; RMSEA = Root Mean Square Error of Approximation; SRMR = Standardized Root Mean Square Residual. Models were estimated using WLSMV with ordered categorical indicators, following the identification approach of Wu and Estabrook (2016). Scaled CFI and RMSEA values are reported for the measurement-invariance analyses because lavaan did not provide robust CFI, TLI, or RMSEA values for the threshold-, loading-, and intercept-constrained multigroup models. For the age analysis, categories 6 and 7 were collapsed for items 8 and 16. For the education analysis, categories 5-7 were collapsed for item 4, and categories 6 and 7 were collapsed for items 6, 8, 10, and 13. No category collapsing was required for the gender analysis. | | | | |

| **Table S22. Changes in fit indices across levels of ordinal measurement invariance (three-factor model, WLSMV)** | | | | |  |
| --- | --- | --- | --- | --- | --- |
|  |  | Δ**CFI** | Δ**RMSEA** | Δ**SRMR** | |
| Age group | Configural → threshold | -.0027 | -.0127 | < .0001 | |
|  | Threshold → + loading | .0031 | -.0049 | .0010 | |
|  | + Loading → + intercept | .0027 | -.0043 | .0001 | |
| Gender | Configural → threshold | -.0011 | -.0089 | < .0001 | |
|  | Threshold → + loading | .0035 | -.0047 | .0001 | |
|  | + Loading → + intercept | .0021 | -.0033 | .0001 | |
| Education | Configural → threshold | -.0050 | -.0093 | < .0001 | |
|  | Threshold → + loading | .0043 | -.0058 | .0007 | |
|  | + Loading → + intercept | .0009 | -.0027 | < .0001 | |
| **Note.** Group sizes: Age: < 30 years n = 356; 30-39 years n = 367; 40-49 years n = 399; 50-59 years n = 454; 60-69 years n = 452; ≥ 70 years n = 340 (N = 2,368). Gender: men n = 1059; women n = 1306 (N = 2365; three participants with diverse gender were excluded). Education: basic school graduation n = 549; without graduation n = 60; middle maturity n = 989; technical school n = 99; A-level n = 357; university graduation n = 307 (N = 2361). Changes in fit were calculated from unrounded scaled values. Threshold invariance was tested before equality constraints were imposed on factor loadings and intercepts, following Wu and Estabrook (2016). As primary criteria, invariance was considered supported when the decrease in CFI did not exceed .010 and the increase in RMSEA did not exceed .015 (Cheung & Rensvold, 2002; Chen, 2007). Given the highly unequal education-group sizes and the small size of two groups, the more stringent change-in-fit criteria discussed by Chen (2007) were additionally considered as a conservative sensitivity benchmark. | | | | | |

# 6 Regression

| **Table S23. Model summaries for the EBQ total score and subscales** | | | | | | | |
| --- | --- | --- | --- | --- | --- | --- | --- |
| **Outcome** | **N** | **R²** | **Adjusted R²** | **F** | **df1** | **df2** | **p** |
| EBQ total score | 2,335 | .060 | .054 | 10.53 | 14 | 2320 | < .001 |
| Controllability | 2,335 | .044 | .039 | 7.71 | 14 | 2320 | < .001 |
| Negative Usefulness | 2,335 | .037 | .031 | 6.29 | 14 | 2320 | < .001 |
| Positive Usefulness | 2,335 | .045 | .039 | 7.84 | 14 | 2320 | < .001 |

**Table S24. Complete sociodemographic regression results (Panels A-D)**

**Panel A. EBQ total score**

| **Predictor** | **B** | **SE** | **95% CI for B** | **Standardized coefficient** | **95% CI standardized** | **t** | **p** | **p_FDR** |
| --- | --- | --- | --- | --- | --- | --- | --- | --- |
| Age (centered) | 0.068 | 0.02 | [0.02, 0.11] | .08 | [.02, .13] | 2.86 | .004 | .013 |
| Gender: Women (ref. Men) | -1.794 | 0.63 | [-3.04, -0.55] | -.12 | [-.20, -.04] | -2.83 | .005 | .013 |
| Education: Without graduation | -0.219 | 2.06 | [-4.25, 3.81] | -.01 | [-.28, .25] | -0.11 | .915 | .915 |
| Education: Middle maturity | -3.266 | 0.84 | [-4.92, -1.62] | -.21 | [-.32, -.11] | -3.88 | < .001 | < .001 |
| Education: Technical school | -3.580 | 1.68 | [-6.87, -0.29] | -.23 | [-.45, -.02] | -2.13 | .033 | .077 |
| Education: A-level | -7.570 | 1.09 | [-9.71, -5.43] | -.49 | [-.63, -.35] | -6.93 | < .001 | < .001 |
| Education: University graduation | -8.566 | 1.12 | [-10.76, -6.37] | -.56 | [-.70, -.42] | -7.64 | < .001 | < .001 |
| Income: €1250-<€2500 | -1.642 | 1.16 | [-3.92, 0.63] | -.11 | [-.26, .04] | -1.42 | .157 | .275 |
| Income: >€2500 | -2.207 | 1.21 | [-4.57, 0.16] | -.14 | [-.30, .01] | -1.83 | .067 | .135 |
| Marital status: Married (living without partner) | 0.399 | 2.16 | [-3.83, 4.63] | .03 | [-.25, .30] | 0.18 | .853 | .915 |
| Marital status: Unmarried (in a relationship) | 0.780 | 1.14 | [-1.47, 3.02] | .05 | [-.10, .20] | 0.68 | .496 | .631 |
| Marital status: Unmarried (not in a relationship) | -0.185 | 1.04 | [-2.23, 1.86] | -.01 | [-.15, .12] | -0.18 | .859 | .915 |
| Marital status: Divorced | -1.199 | 1.03 | [-3.21, 0.81] | -.08 | [-.21, .05] | -1.17 | .243 | .378 |
| Marital status: Widowed | 1.360 | 1.31 | [-1.21, 3.92] | .09 | [-.08, .26] | 1.04 | .299 | .418 |

**Panel B. Controllability**

| **Predictor** | **B** | **SE** | **95% CI for B** | **Standardized coefficient** | **95% CI standardized** | **t** | **p** | **p_FDR** |
| --- | --- | --- | --- | --- | --- | --- | --- | --- |
| Age (centered) | 0.032 | 0.01 | [0.00, 0.06] | .06 | [.01, .11] | 2.19 | .029 | .057 |
| Gender: Women (ref. Men) | -1.168 | 0.39 | [-1.94, -0.40] | -.12 | [-.21, -.04] | -2.98 | .003 | .010 |
| Education: Without graduation | -0.753 | 1.27 | [-3.24, 1.74] | -.08 | [-.35, .19] | -0.59 | .553 | .646 |
| Education: Middle maturity | -2.006 | 0.52 | [-3.03, -0.99] | -.21 | [-.32, -.11] | -3.86 | < .001 | < .001 |
| Education: Technical school | -2.642 | 1.04 | [-4.68, -0.61] | -.28 | [-.50, -.06] | -2.55 | .011 | .027 |
| Education: A-level | -3.949 | 0.68 | [-5.27, -2.62] | -.42 | [-.56, -.28] | -5.85 | < .001 | < .001 |
| Education: University graduation | -4.229 | 0.69 | [-5.59, -2.87] | -.45 | [-.60, -.31] | -6.11 | < .001 | < .001 |
| Income: €1250-<€2500 | -1.531 | 0.72 | [-2.94, -0.13] | -.16 | [-.31, -.01] | -2.14 | .033 | .057 |
| Income: >€2500 | -1.879 | 0.74 | [-3.34, -0.42] | -.20 | [-.36, -.04] | -2.52 | .012 | .027 |
| Marital status: Married (living without partner) | 0.379 | 1.33 | [-2.23, 2.99] | .04 | [-.24, .32] | 0.28 | .776 | .814 |
| Marital status: Unmarried (in a relationship) | 0.814 | 0.71 | [-0.57, 2.20] | .09 | [-.06, .23] | 1.15 | .250 | .350 |
| Marital status: Unmarried (not in a relationship) | -0.152 | 0.64 | [-1.41, 1.11] | -.02 | [-.15, .12] | -0.24 | .814 | .814 |
| Marital status: Divorced | -0.549 | 0.63 | [-1.79, 0.69] | -.06 | [-.19, .07] | -0.87 | .387 | .492 |
| Marital status: Widowed | 1.006 | 0.81 | [-0.58, 2.59] | .11 | [-.06, .28] | 1.24 | .213 | .332 |

**Panel C. Negative Usefulness**

| **Predictor** | **B** | **SE** | **95% CI for B** | **Standardized coefficient** | **95% CI standardized** | **t** | **p** | **p_FDR** |
| --- | --- | --- | --- | --- | --- | --- | --- | --- |
| Age (centered) | 0.016 | 0.01 | [0.00, 0.03] | .05 | [.00, .10] | 1.80 | .072 | .257 |
| Gender: Women (ref. Men) | -0.290 | 0.23 | [-0.75, 0.17] | -.05 | [-.13, .03] | -1.24 | .214 | .344 |
| Education: Without graduation | -1.142 | 0.76 | [-2.63, 0.34] | -.20 | [-.47, .06] | -1.51 | .132 | .263 |
| Education: Middle maturity | -0.550 | 0.31 | [-1.16, 0.06] | -.10 | [-.21, .01] | -1.77 | .077 | .257 |
| Education: Technical school | -0.693 | 0.62 | [-1.91, 0.52] | -.12 | [-.34, .09] | -1.12 | .262 | .344 |
| Education: A-level | -1.890 | 0.40 | [-2.68, -1.10] | -.34 | [-.48, -.20] | -4.69 | < .001 | < .001 |
| Education: University graduation | -2.538 | 0.41 | [-3.35, -1.73] | -.46 | [-.60, -.31] | -6.15 | < .001 | < .001 |
| Income: €1250-<€2500 | 0.709 | 0.43 | [-0.13, 1.55] | .13 | [-.02, .28] | 1.66 | .097 | .257 |
| Income: >€2500 | 0.349 | 0.44 | [-0.52, 1.22] | .06 | [-.09, .22] | 0.79 | .432 | .465 |
| Marital status: Married (living without partner) | -0.738 | 0.79 | [-2.30, 0.82] | -.13 | [-.41, .15] | -0.93 | .353 | .412 |
| Marital status: Unmarried (in a relationship) | -0.674 | 0.42 | [-1.50, 0.15] | -.12 | [-.27, .03] | -1.60 | .110 | .257 |
| Marital status: Unmarried (not in a relationship) | -0.423 | 0.38 | [-1.17, 0.33] | -.08 | [-.21, .06] | -1.10 | .271 | .344 |
| Marital status: Divorced | -0.256 | 0.38 | [-1.00, 0.49] | -.05 | [-.18, .09] | -0.68 | .499 | .499 |
| Marital status: Widowed | -0.570 | 0.48 | [-1.51, 0.38] | -.10 | [-.27, .07] | -1.18 | .237 | .344 |

**Panel D. Positive Usefulness**

| **Predictor** | **B** | **SE** | **95% CI for B** | **Standardized coefficient** | **95% CI standardized** | **t** | **p** | **p_FDR** | |
| --- | --- | --- | --- | --- | --- | --- | --- | --- | --- |
| Age (centered) | 0.020 | 0.01 | [0.01, 0.03] | .07 | [.02, .13] | 2.76 | .006 | .022 | |
| Gender: Women (ref. Men) | -0.335 | 0.20 | [-0.72, 0.05] | -.07 | [-.15, .01] | -1.72 | .086 | .120 | |
| Education: Without graduation | 1.676 | 0.63 | [0.44, 2.92] | .36 | [.09, .62] | 2.65 | .008 | .023 | |
| Education: Middle maturity | -0.710 | 0.26 | [-1.22, -0.20] | -.15 | [-.26, -.04] | -2.74 | .006 | .022 | |
| Education: Technical school | -0.244 | 0.52 | [-1.26, 0.77] | -.05 | [-.27, .16] | -0.47 | .636 | .636 | |
| Education: A-level | -1.731 | 0.34 | [-2.39, -1.07] | -.37 | [-.51, -.23] | -5.15 | < .001 | < .001 | |
| Education: University graduation | -1.799 | 0.34 | [-2.47, -1.12] | -.38 | [-.53, -.24] | -5.22 | < .001 | < .001 | |
| Income: €1250-<€2500 | -0.820 | 0.36 | [-1.52, -0.12] | -.18 | [-.33, -.03] | -2.30 | .022 | .044 | |
| Income: >€2500 | -0.677 | 0.37 | [-1.40, 0.05] | -.14 | [-.30, .01] | -1.83 | .068 | .107 | |
| Marital status: Married (living without partner) | 0.758 | 0.66 | [-0.54, 2.06] | .16 | [-.12, .44] | 1.14 | .253 | .273 | |
| Marital status: Unmarried (in a relationship) | 0.640 | 0.35 | [-0.05, 1.33] | .14 | [-.01, .28] | 1.82 | .069 | .107 | |
| Marital status: Unmarried (not in a relationship) | 0.389 | 0.32 | [-0.24, 1.02] | .08 | [-.05, .22] | 1.22 | .224 | .262 | |
| Marital status: Divorced | -0.394 | 0.32 | [-1.01, 0.22] | -.08 | [-.22, .05] | -1.25 | .212 | .262 | |
| Marital status: Widowed | 0.923 | 0.40 | [0.13, 1.71] | .20 | [.03, .37] | 2.29 | .022 | .044 | |
| Note. B = unstandardized regression coefficient; CI = confidence interval. For age, the standardized coefficient was calculated as B × SDX / SDY; for categorical predictors, it represents an outcome-standardized dummy-coded contrast (B / SDY). p_FDR denotes Benjamini-Hochberg adjusted p values across the 14 non-intercept coefficients within the respective outcome. Reference categories were men, basic school graduation, household income < €1250, and married and living with partner. Age was mean-centered at M = 50.42 years. | | | | | | | | |  |

| **Table S25. Omnibus predictor-block tests and unique effect sizes for EBQ total and subscale scores** | | | | | | | | |
| --- | --- | --- | --- | --- | --- | --- | --- | --- |
| **Outcome** | **Predictor** | **df1** | **df2** | **F** | **p** | **p_FDR** | **Partial R²** | **ΔR²** |
| EBQ total score | Age | 1 | 2320 | 8.21 | .004 | .008 | .004 | .003 |
| EBQ total score | Gender | 1 | 2320 | 8.00 | .005 | .008 | .003 | .003 |
| EBQ total score | Education | 5 | 2320 | 16.25 | < .001 | < .001 | .034 | .033 |
| EBQ total score | Income | 2 | 2320 | 1.67 | .188 | .235 | .001 | .001 |
| EBQ total score | Marital status | 5 | 2320 | 0.88 | .493 | .493 | .002 | .002 |
| Controllability | Age | 1 | 2320 | 4.80 | .029 | .048 | .002 | .002 |
| Controllability | Gender | 1 | 2320 | 8.89 | .003 | .007 | .004 | .004 |
| Controllability | Education | 5 | 2320 | 10.33 | < .001 | < .001 | .022 | .021 |
| Controllability | Income | 2 | 2320 | 3.22 | .040 | .0499 | .003 | .003 |
| Controllability | Marital status | 5 | 2320 | 1.09 | .366 | .366 | .002 | .002 |
| Negative Usefulness | Age | 1 | 2320 | 3.23 | .072 | .181 | .001 | .001 |
| Negative Usefulness | Gender | 1 | 2320 | 1.55 | .214 | .267 | .001 | .001 |
| Negative Usefulness | Education | 5 | 2320 | 10.28 | < .001 | < .001 | .022 | .021 |
| Negative Usefulness | Income | 2 | 2320 | 1.68 | .187 | .267 | .001 | .001 |
| Negative Usefulness | Marital status | 5 | 2320 | 0.83 | .528 | .528 | .002 | .002 |
| Positive Usefulness | Age | 1 | 2320 | 7.61 | .006 | .015 | .003 | .003 |
| Positive Usefulness | Gender | 1 | 2320 | 2.95 | .086 | .086 | .001 | .001 |
| Positive Usefulness | Education | 5 | 2320 | 11.47 | < .001 | < .001 | .024 | .024 |
| Positive Usefulness | Income | 2 | 2320 | 2.64 | .071 | .086 | .002 | .002 |
| Positive Usefulness | Marital status | 5 | 2320 | 2.98 | .011 | .018 | .006 | .006 |
| **Note.** Omnibus tests evaluate each complete predictor block after adjustment for all other predictors in the model. p_FDR denotes Benjamini-Hochberg adjusted p values across the five omnibus predictor-block tests within each outcome. Partial R² represents the proportion of residual variance uniquely attributable to the tested predictor block, whereas ΔR² represents the unique increment in total explained outcome variance associated with that block. | | | | | | | | |

| **Table S26. Bivariate associations between age and EBQ outcomes** | | | |
| --- | --- | --- | --- |
| **Outcome** | **r** | **95% CI** | **p** |
| EBQ total score | .126 | [.086, .166] | < .001 |
| Controllability | .103 | [.063, .143] | < .001 |
| Negative Usefulness | .092 | [.051, .132] | < .001 |
| Positive Usefulness | .096 | [.056, .136] | < .001 |
| **Note**. r denotes Pearson’s correlation coefficient; CI = confidence interval. | | | |

| **Table S27. Bivariate omnibus associations for categorical sociodemographic variables** | | | | | | |
| --- | --- | --- | --- | --- | --- | --- |
| **Outcome** | **Predictor** | **F** | **df1** | **df2** | **p** | **Bivariate R²** |
| EBQ total score | Gender | 5.16 | 1 | 2333 | .023 | .002 |
| EBQ total score | Education | 23.78 | 5 | 2329 | < .001 | .049 |
| EBQ total score | Income | 8.52 | 2 | 2332 | < .001 | .007 |
| EBQ total score | Marital status | 4.88 | 5 | 2329 | < .001 | .010 |
| Controllability | Gender | 6.25 | 1 | 2333 | .013 | .003 |
| Controllability | Education | 16.00 | 5 | 2329 | < .001 | .033 |
| Controllability | Income | 8.99 | 2 | 2332 | < .001 | .008 |
| Controllability | Marital status | 3.79 | 5 | 2329 | .002 | .008 |
| Negative Usefulness | Gender | 0.91 | 1 | 2333 | .340 | .000 |
| Negative Usefulness | Education | 14.07 | 5 | 2329 | < .001 | .029 |
| Negative Usefulness | Income | 5.45 | 2 | 2332 | .004 | .005 |
| Negative Usefulness | Marital status | 3.53 | 5 | 2329 | .003 | .008 |
| Positive Usefulness | Gender | 1.65 | 1 | 2333 | .198 | .001 |
| Positive Usefulness | Education | 15.64 | 5 | 2329 | < .001 | .032 |
| Positive Usefulness | Income | 6.60 | 2 | 2332 | .001 | .006 |
| Positive Usefulness | Marital status | 5.18 | 5 | 2329 | < .001 | .011 |

| **Table S28. Regression diagnostics across EBQ outcomes** | | | | | | |
| --- | --- | --- | --- | --- | --- | --- |
| **Outcome** | **Max adjusted GVIF** | **Breusch-Pagan p** | **Quadratic age p** | **ΔR² quadratic age** | **Max Cook's D** | **\|Standardized residual\| > 3** |
| EBQ total score | 1.31 | .508 | .432 | .0003 | .010 | 6 |
| Controllability | 1.31 | .345 | .814 | .0000 | .008 | 11 |
| Negative Usefulness | 1.31 | < .001 | .076 | .0013 | .009 | 0 |
| Positive Usefulness | 1.31 | .231 | .963 | .0000 | .010 | 14 |
| **Note.** GVIF = generalized variance inflation factor, adjusted for degrees of freedom. Quadratic age p refers to the model comparison including an additional squared age term. No case exceeded conventional Cook's distance thresholds (all D < .50). N = 2,335 for all models. | | | | | | |

| **Table S29. Significance decisions differing between primary and sensitivity analyses** | | | | | |
| --- | --- | --- | --- | --- | --- |
| **Comparison** | **Outcome** | **Level** | **Predictor** | **Primary p_FDR** | **Sensitivity p_FDR** |
| OLS vs. HC3 | Controllability | Coefficient | Age (centered) | .057 | .046 |
| OLS vs. HC3 | Positive Usefulness | Coefficient | Income: €1250-<€2500 | .044 | .063 |
| OLS vs. HC3 | Positive Usefulness | Coefficient | Marital status: Widowed | .044 | .063 |
| OLS vs. HC3 | Controllability | Predictor block | Income | .0499 | .084 |
| Outcome-specific vs. pooled FDR | Positive Usefulness | Coefficient | Income: €1250-<€2500 | .044 | .061 |
| Outcome-specific vs. pooled FDR | Positive Usefulness | Coefficient | Marital status: Widowed | .044 | .061 |
| Outcome-specific vs. pooled FDR | Controllability | Predictor block | Age | .048 | .057 |
| Outcome-specific vs. pooled FDR | Controllability | Predictor block | Income | .0499 | .073 |
| **Note.** The table lists all cases in which the statistical significance decision (p_FDR < .05) differed between the primary analysis and a sensitivity analysis. Across the 152 paired decisions examined (76 comparisons using HC3 heteroskedasticity-consistent standard errors and 76 comparisons using pooled FDR correction), 8 differed and 144 were concordant. The primary analysis used ordinary least squares with Benjamini-Hochberg FDR correction applied separately within each outcome. Sensitivity analyses used either HC3 heteroskedasticity-consistent standard errors or Benjamini-Hochberg correction across all four outcomes jointly, as indicated. Adjusted p values are reported to four decimal places where rounding to three would obscure the direction of the decision. All divergent findings were close to the .05 significance threshold. | | | | | |
